# CaRuby-Nano: a novel high affinity calcium probe for dual color imaging

**DOI:** 10.7554/eLife.05808

**Published:** 2015-03-31

**Authors:** Mayeul Collot, Christian D Wilms, Asma Bentkhayet, Païkan Marcaggi, Kiri Couchman, Serge Charpak, Stéphane Dieudonné, Michael Häusser, Anne Feltz, Jean-Maurice Mallet

**Affiliations:** 1 Laboratory of Biomolecules, UPMC Université Paris 06, Ecole Normale Supérieure, Paris, France; 2 Wolfson Institute for Biomedical Research and Department of Neuroscience, Physiology and Pharmacology, University College London, London, United Kingdom; 3 Institut de Biologie de l'École Normale Supérieure, CNRS UMR8197, INSERM U1024, Paris, France; 4 Institut National de la Santé et de la Recherche Médicale, Paris, France; 5 Laboratory of Neurophysiology and New Microscopies, Université Paris Descartes, Paris, France; Northwestern University, United States

**Keywords:** calcium, two-photon imaging, click chemistry, fluorescence, mouse

## Abstract

The great demand for long-wavelength and high signal-to-noise Ca^2+^ indicators has led us to develop CaRuby-Nano, a new functionalizable red calcium indicator with nanomolar affinity for use in cell biology and neuroscience research. In addition, we generated CaRuby-Nano dextran conjugates and an AM-ester variant for bulk loading of tissue. We tested the new indicator using in vitro and in vivo experiments demonstrating the high sensitivity of CaRuby-Nano as well as its power in dual color imaging experiments.

**DOI:** http://dx.doi.org/10.7554/eLife.05808.001

## eLife digest

The movement of calcium ions within cells controls many vital biological processes, ranging from cell growth to muscle contraction and brain activity. These calcium signals are triggered by stimuli, such as nerve impulses, which drive calcium entry into cells or release calcium from internal stores. These changes in calcium levels can span several orders of magnitude, and can be either localized to very small parts of the cell or span the entire cell.

Scientists have developed numerous indicators or ‘probes’ that can detect even very low levels of calcium. One common method uses proteins that fluoresce when viewed under a fluorescence microscope each time the protein senses increases of calcium. Most of these probes fluoresce green, and so to view a second signal that occurs in the cell at the same time it's easier to use a probe that fluoresces with a different color, such as red. However, the red-shifted probes that are currently available either produce unreliable results because they tend to leak through cell membranes, or are not very sensitive to calcium ions. New types of red-shifted probes are therefore urgently needed.

In 2012, researchers developed a family of red fluorescent probes known as Calcium Ruby (CaRuby for short) that were more versatile than earlier red probes. Now, Collot, Wilms et al.—including several of the researchers involved in the 2012 research—have enhanced the properties of CaRuby by modifying the chemical structure of the probes. This increased the ability of CaRuby to bind calcium ions, making it more sensitive to small calcium changes. Testing the usefulness of the newly developed probes—called CaRuby Nano—in mouse nerve cells revealed the probes are highly sensitive and can even detect the calcium signal resulting from a single nerve impulse.

Collot, Wilms et al. then went on to demonstrate that CaRuby-Nano can be used alongside a green-fluorescing probe to record two signals at the same time. In one experiment, the release of chemical messengers known as neurotransmitters was stimulated, which caused calcium ions to flow into the observed nerve cells. The researchers succeeded in simultaneously detecting a green signal indicating an increase in neurotransmitter levels and a red signal produced by the corresponding release of calcium. Such dual-color imaging was not possible with previous probes. Finally, it was shown that CaRuby-Nano can also be used to produce dual-color images of the brain activity of live mice.

In summary, these results demonstrate that CaRuby-Nano is a highly sensitive and versatile indicator and can be used together with other probes to observe two simultaneous events in cells.

**DOI:** http://dx.doi.org/10.7554/eLife.05808.002

## Introduction

In recent years fluorescence imaging has been one of the fastest growing methods in physiology, cell biology and neuroscience, constantly driving the need for improved fluorescent probes (Wilt et al., 2009; Miyawaki, 2011; Looger and Griesbeck, 2012). The dominance of fluorescein and eGFP in the design of such probes has resulted in an overcrowding of the green spectral band. This makes simultaneous imaging of spatially overlapping signals problematic and emphasizes the need for red-shifted probes (Oheim et al., 2014). Many of the very favorable photophysical properties of fluorescein and eGFP are shared by the X-Rhodamine chromophore, which is finding increasing use in the development of Ca^2+^-indicators (Eberhard and Erne, 1991; Egawa et al., 2011). A major problem of red-shifted fluorophores is that they are significantly more lipophilic than fluorescein-like dyes. This leads to more leakage through cell membranes as well as to intracellular compartmentalization. These effects can be minimized by using the probes as conjugates of inert hydrophilic compounds such as dextrans. This conjugation commonly uses one of the carboxylic groups of the BAPTA moiety, affecting the calcium affinity of BAPTA-based indicators (Tsien, 1980). Consequently, these indicator-dextran conjugates are strongly shifted to lower affinities making them all but useless for sensitive and quantitative [Ca^2+^] measurements. Importantly, this lower affinity cannot be compensated for by increasing the concentration of the probe. Such a strategy would lead to a disproportionally large increase in the calcium buffering capacity of the indicator (Neher, 2005), resulting in a stronger disruption of cellular signaling than for a low concentration of high affinity indicator (Markram et al., 1998).

We recently introduced a family of red emitting calcium indicators based on X-Rhodamine: Calcium Ruby (CaRuby) (Collot et al., 2012), which bears an azido side arm for click chemistry and the resulting potential for high-yield coupling reactions (Kolb et al., 2001). This side arm efficiently allows conjugation reactions without significant perturbation of the calcium binding affinity (Zamaleeva et al., 2014). The dissociation constants of CaRubies ranged from 3.4 to 21.6 µM—too high for the reliable detection of small [Ca^2+^] transients in biological tissue (Yasuda et al., 2004) (see Appendix 1 for details).

## Results

**Figure 1.**
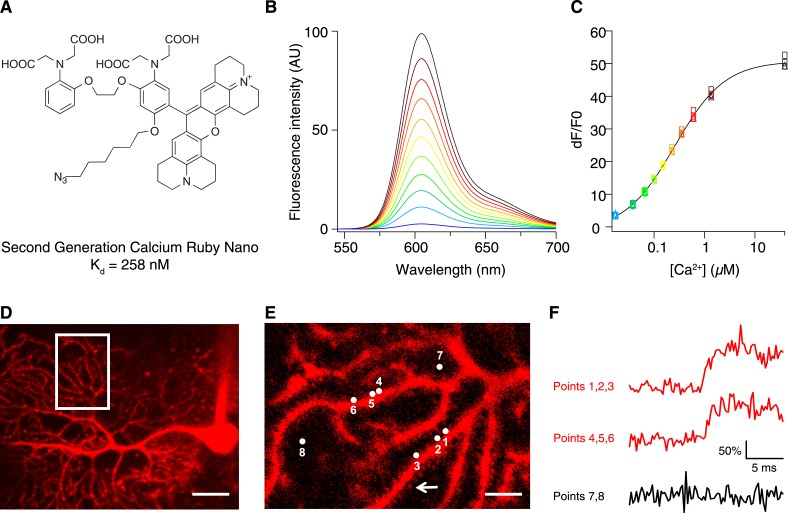
Chemical and photophysical properties of CaRuby-Nano. (**A**) Structure of CaRuby-Nano. Note the oxygen substituent and the positioning of the fluorophore-BAPTA bond. (**B**) [Ca^2+^]-dependent change in CaRuby-Nano fluorescence ([Ca^2+^]_free_: 0 nM, 17 nM, 38 nM, 65 nM, 100 nM, 150 nM, 225 nM, 351 nM, 602 nM, 1.35 µM, 39 µM). (**C**) The titration curve corresponding to the spectra in (**B**) using the same color code. (**D**–**F**): Climbing fiber evoked dendritic calcium signals in Purkinje cells in vitro. (**D**) Purkinje cell filled with 300 µM CaRuby-Nano dextran, with region of interest indicated by the white rectangle (scale bar = 20 µm). (**E**) Region of interest with points of interest indicated. Note that many spines can be readily distinguished (white arrow). Points 1–3 and 4–6 are on different spiny branchlets while points 7 and 8 are background (scale bar = 5 µm). (**F**) Ca^2+^ transients following climbing fiber activation recorded at 2.8 kHz (traces averaged over 26 trials and then averaged over the indicated spine numbers). **DOI:** http://dx.doi.org/10.7554/eLife.05808.003

To increase the affinity of CaRuby, we modified the structure of the probe (Figure 1A), focusing on the Ca^2+^ chelating BAPTA moiety, as increasing the electron density of BAPTA lowers its K_D_ for calcium (Tsien, 1980). We introduced an oxygen atom on one of the aromatic rings of BAPTA by a S_N_Ar reaction. This oxygen also serves as a link for the azido side arm, which was repositioned in the new CaRuby variant (Figure 1—figure supplement 1). Additionally, the fluorophore, which is commonly placed *para* to the nitrogen of the BAPTA, has an affinity-lowering effect due to its electron withdrawing nature and was therefore placed at a *meta* position in order to reduce its effect on the ligating nitrogen. These modifications resulted in a CaRuby variant with sub-micromolar affinity (‘CaRuby-Nano’). In cuvette calibration experiments CaRuby-Nano was found to have a K_D_ of 258 ± 8 nM, with a 50-fold (±2) increase of fluorescence on binding [Ca^2+^] (Figure 1B–C) and a maximum quantum yield of 0.45 (Figure 1—figure supplement 2,3). In addition to being suitable for single photon excitation, CaRuby-Nano also exhibits effective two-photon excitation over a large wavelength band (Figure 1—figure supplement 4).

**Figure 1—figure supplement 1.**
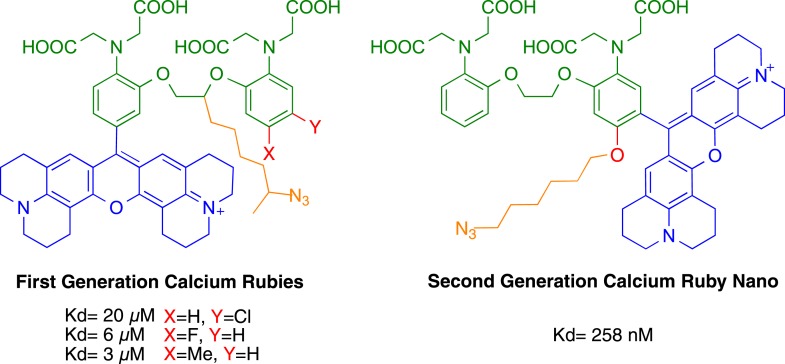
Comparison of CaRuby structures. **DOI:** http://dx.doi.org/10.7554/eLife.05808.004

**Figure 1—figure supplement 2.**
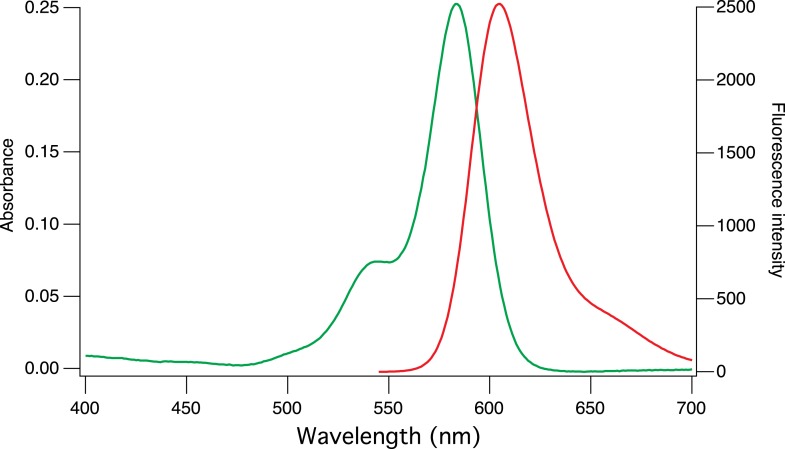
Absorption and emission spectra of CaRu-Nano. **DOI:** http://dx.doi.org/10.7554/eLife.05808.005

**Figure 1—figure supplement 3.**
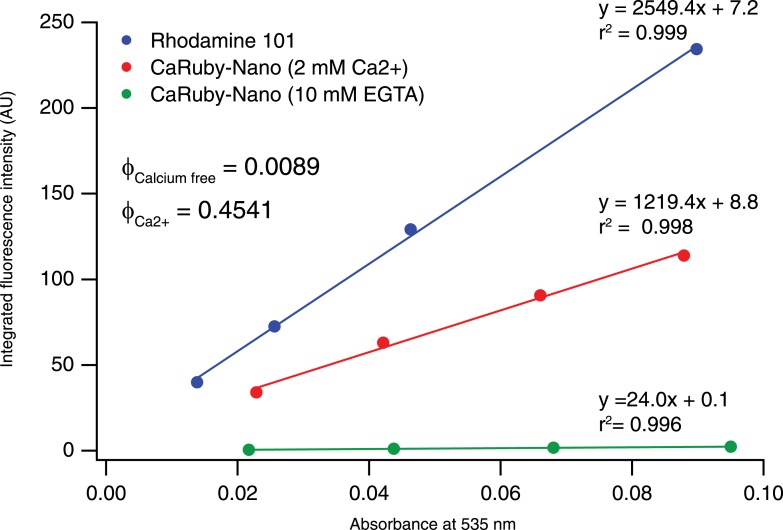
Determination of CaRuby-Nano fluorescence quantum yield. **DOI:** http://dx.doi.org/10.7554/eLife.05808.006

**Figure 1—figure supplement 4.**
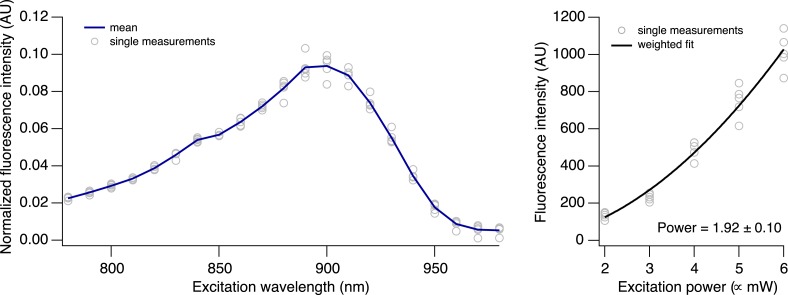
Two-photon excitation of CaRuby-Nano. **DOI:** http://dx.doi.org/10.7554/eLife.05808.007

**Figure 1—figure supplement 5.**
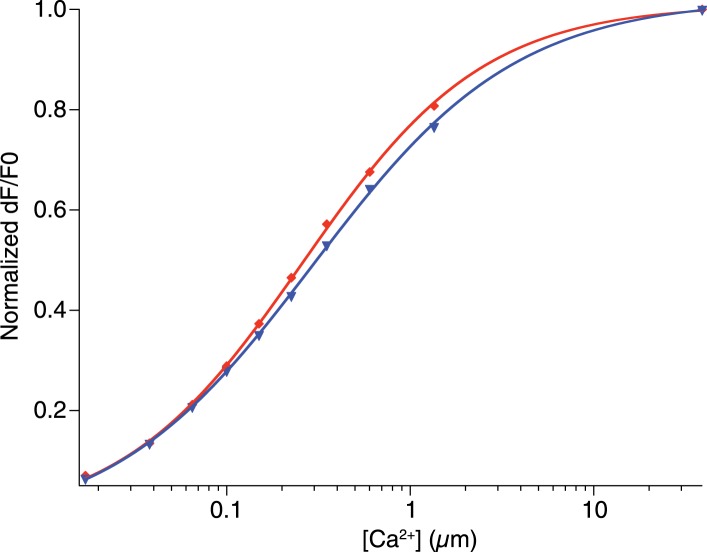
Affinity of CaRuby-Nano and CaRuby-Nano 6 kD dextran. **DOI:** http://dx.doi.org/10.7554/eLife.05808.008

For verification of the new probe in biological tissue we used conjugates with 1.5 kD and 6 kD dextrans, which were obtained via click chemistry. As expected, this conjugation had only a small effect on the affinity of the indicator, increasing the K_D_ from 258 nM in the free salt to 295 nM in the 6 kD dextran conjugate (Figure 1—figure supplement 5).

We first tested if CaRuby-Nano performs comparably to commonly used green emitting [Ca^2+^] probes. For this, Purkinje cells in acute cerebellar brain slices were filled with CaRuby-Nano (1.5 kD dextran) via a patch-clamp microelectrode (Figure 1D). Climbing fiber stimulation evoked calcium signals were recorded from multiple spines (range: 4 to 14) at acquisition rates between 2.2 and 5.0 kHz (Figure 1E,F) using random access two-photon microscopy (Otsu et al., 2008). The rising phase time course (0.55 ms ± 0.13 ms; sigmoidal fit; n = 59 spines from 7 cells) was not significantly different from that found for Fluo-5F (0.40 ± 0.09 ms, n = 36 spines from 4 cells, p = 0.37) under the same conditions, suggesting that CaRuby-Nano has binding kinetics comparable to established small molecule Ca^2+^ indicators. These fast kinetics point to a high sensitivity of CaRuby-Nano for small and fast changes in [Ca^2+^], such as neuronal action potentials (Otsu et al., 2014).

**Figure 2.**
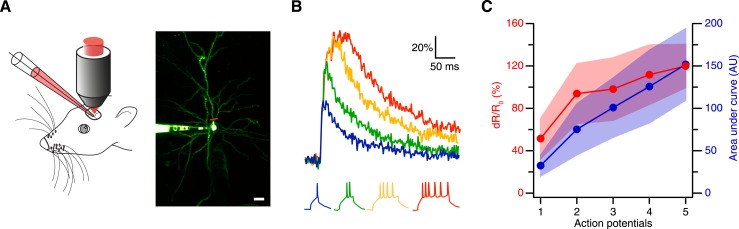
Spike evoked transients in layer 2/3 pyramidal neurons in vivo. (**A**) Measurement configuration (left) and maximum intensity projection of pyramidal neuron filled with 100 µM Alexa Fluor 488 and 200 µM CaRuby-Nano dextran (right, at rest the fluorescence is dominated by the green dye). The red line indicates region imaged in line scan. Scale bar: 20 µm. (**B**) Single trial calcium signals evoked by increasing number of spikes. The corresponding membrane voltage traces are shown below. Fluorescence traces are aligned to spike onset and color-coded to match the number of APs. (**C**) The peak amplitudes (red) and the area under the curve (blue) of the fluorescence trace were plotted against the number of action potentials. While the area increases linearly, the peak amplitude saturates. The shaded regions indicate the corresponding standard deviations. **DOI:** http://dx.doi.org/10.7554/eLife.05808.009

Thus, we next tested the sensitivity of CaRuby-Nano using in vivo patch-clamp recordings from neocortical layer 2/3 pyramidal neurons in anesthetized mice with simultaneous two-photon [Ca^2+^] imaging (Svoboda et al., 1997) (Figure 2A). We found that using CaRuby-Nano (6 kD dextran) even single spikes resulted in reliable, easily detected fluorescence transients (mean dR/R_0_ = 0.52 ± 0.19, n = 6 cells; Figure 2B). For increasing spike numbers the dR/R_0_ vs spike number relation quickly turns sublinear and saturates as expected for high affinity indicators (Figure 2C). Taken together these experiments demonstrate that CaRuby-Nano is a calcium indicator with a signal quality comparable to previously used high-affinity green emitting probes. Importantly, it is well suited for the detection of small [Ca^2+^] transients, setting it apart from the previous CaRuby versions.

**Figure 3.**
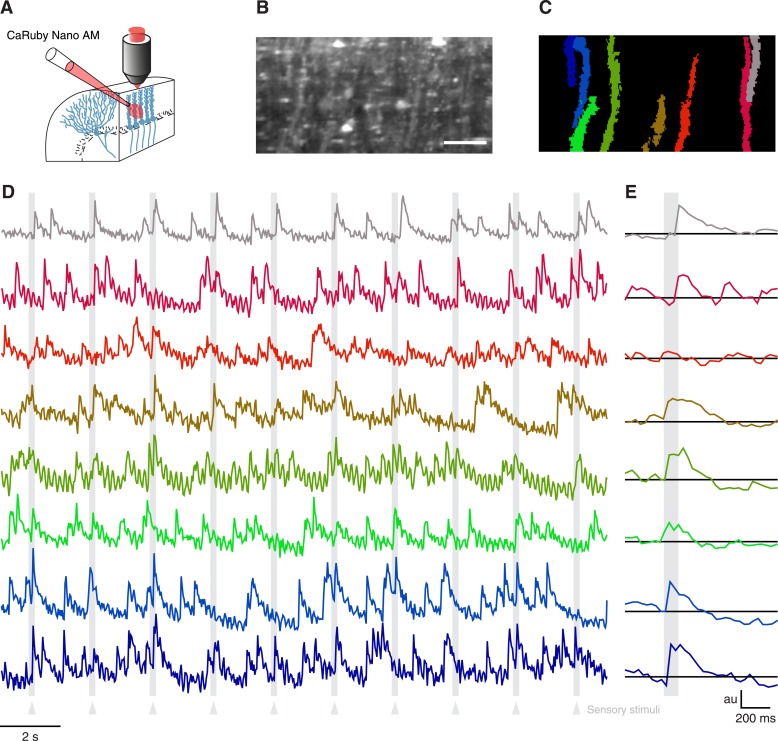
Imaging cerebellar Purkinje cells in vivo using bulk loading of CaRuby-Nano AM. (**A**) Configuration of AM-ester injection and imaging. (**B**) Resulting staining of tissue 60 min after injection of indicator. Purkinje cells can be seen as vertical stripes with occasional brighter spots (corresponding to dendrites; scale bar = 20 µm). (**C**) Active Purkinje cell dendrites identified using a spatial PCA/ICA algorithm (Ozden et al., 2009). (**D**) Fluorescence traces from the identified dendrites, using the same color code as in (**C**). The timing of the sensory stimulus (foot shock) is indicated by the underlying grey bars. (**E**) Stimulus triggered averages of 20 stimulus presentations. Note that all cells except for the third (orange) show a stimulus-locked response. **DOI:** http://dx.doi.org/10.7554/eLife.05808.010

Having verified the suitability of CaRuby-Nano for single cell imaging experiments in vitro and in vivo, we now set out to test CaRuby-Nano for imaging neuronal network activity when applying sensory stimulation. In the past decade calcium population imaging has commonly been performed using bulk loading (Stosiek et al., 2003; Ohki et al., 2005) of calcium indicators in the AM-ester form (Tsien, 1981). We thus synthesized an AM-ester of CaRuby-Nano and used it to load cerebellar neurons in vivo (Figure 3A). We performed a series of three experiments. In all cases we found labeling identical to that commonly found in experiments using Oregon Green-488 BAPTA-1 AM (OGB-1 AM) to load cerebellar tissue in vivo (Figure 3B) (Sullivan et al., 2005; Ozden et al., 2009; Schultz et al., 2009). In all experiments fluorescence traces extracted from identified Purkinje cell dendrites (Figure 3C) showed clear complex spike activity with a good signal-to-noise ratio (Figure 3D,F). Both spontaneous activity and sensory evoked responses were again comparable to signals detected in experiments using OGB-1 AM (Sullivan et al., 2005; Ozden et al., 2009; Schultz et al., 2009). These results indicate that CaRuby-Nano AM is a powerful addition to the optophysiological toolbox.

**Figure 4.**
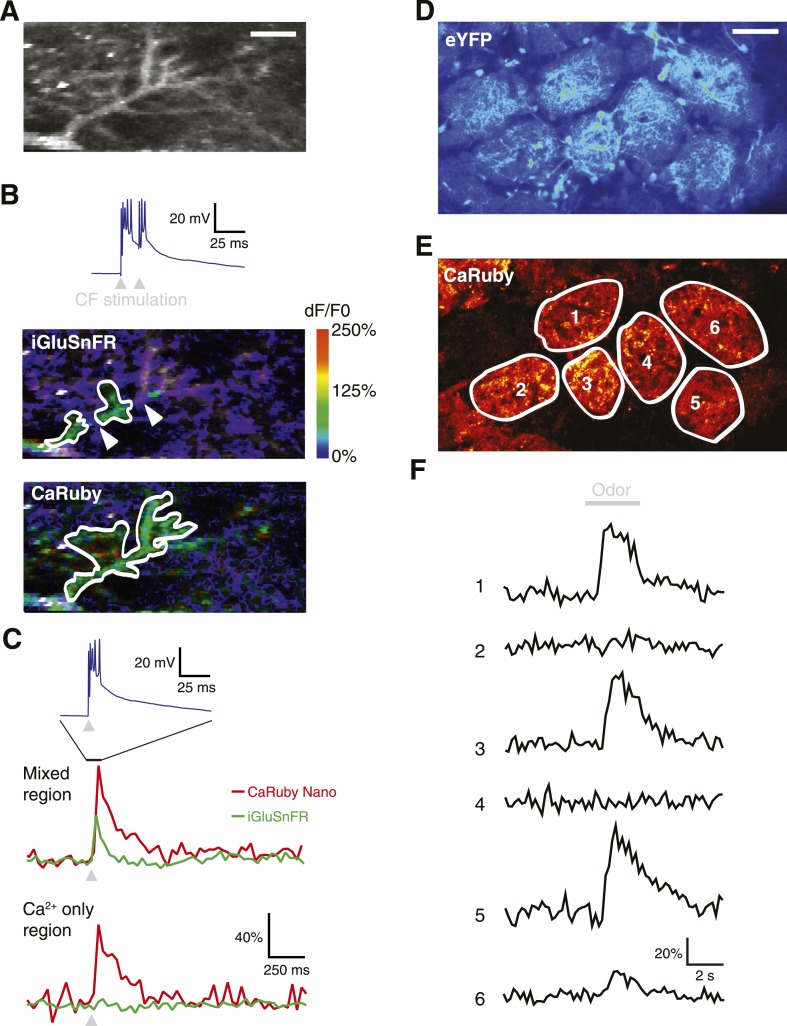
Dual color functional imaging in vitro and in vivo. (**A**–**C**) Combined imaging of [glutamate] and [Ca^2+^] in vitro (**A**) A Purkinje cell expressing iGluSnFR was filled with 200 µM CaRuby-Nano dextran. The image shows the basal fluorescence of CaRuby-Nano (scale bar = 5 µm). (**B**) Double pulse stimulation of the climbing fiber triggers spatially distinct patterns of glutamate release and Ca^2+^ influx (maximum dF/F_0_ images; the inset at the top shows the two evoked complex spikes). Note the breaks between regions showing iGluSnFR activation (indicated by white arrows) (**C**) Fluorescence traces for CaRuby-Nano (red) and iGluSnFR (green) following single pulse climbing fiber stimulation (top inset). Traces were extracted from the corresponding regions outlined in white in (**B**). Note the absence of a fluorescent transient for iGluSnFR in the ‘Ca^2+^ only’ region. (**D**–**F**) Odor-evoked calcium responses in olfactory bulb glomeruli in vivo. (**D**) Juxtaglomerular neurons and mitral cell dendritic tufts expressing YFP demarcate glomeruli in a Kv3.1-eYFP mouse (Metzger et al, 2002). (**E**) Olfactory sensory neuron glutamatergic terminals, labeled with CaRuby-Nano dextran, clearly filled the inner boundaries of most glomeruli. (**F**) A 3 s application of 30% isoamyl acetate reliably triggered presynaptic calcium responses in several glomeruli. **DOI:** http://dx.doi.org/10.7554/eLife.05808.011

To demonstrate the full power of CaRuby-Nano, we made use of the strong two-photon excitation spectral overlap with eGFP to conduct a set of experiments which were not possible previously: simultaneous imaging of glutamate release onto Purkinje cells using iGluSnFR (a single-wavelength extracellular glutamate indicator constructed from the bacterial glutamate sensor Gltl and circularly permutated GFP [Marvin et al., 2013]) and the resulting post-synaptic [Ca^2+^] increase (using CaRuby-Nano). Visually identified Purkinje cells showing iGluSnFR expression (7–9 days after viral transfection) were filled with CaRuby-Nano via patch-clamp recording (6 kD dextran; Figure 4A). Activation of the glutamatergic climbing fiber input evoked clear fluorescence transients in both color channels (Figure 4B). Glutamate signals were confined to distinct subsections of the dendritic tree (i.e., limited to sites of synaptic glutamate release), whereas the resulting [Ca^2+^] transients were global, with similar amplitudes throughout different regions of the dendritic tree (Figure 4C) (Lev-Ram et al., 1992). The differential spatial distribution of the signals confirms that the two indicators can be spectrally isolated.

To demonstrate that dual color imaging is also possible in vivo we used CaRuby-Nano (6 kD dextran) to report presynaptic activity in anesthetized Kv3.1-eYFP adult mice (Metzger et al., 2002). In the olfactory bulb of these mice, mitral and tufted cells, as well as a population of periglomerular neurons, strongly express eYFP and their somata and processes clearly demarcate the external glomerular boundaries (Figure 4D). Olfactory sensory neuron (OSN) terminals, labeled with CaRuby-Nano, filled the inner glomerular boundaries (Figure 4E). In single glomeruli (n = 8 animals) we could record presynaptic calcium responses with excellent signal to noise ratio. Figure 4F shows a typical example in which presynaptic calcium responses were selectively evoked by odor presentation in a subset of glomeruli. These responses adapted strongly at this high odorant concentration, as reported previously (Lecoq et al., 2009). Taken together, these last two experiments demonstrate the potential of two-channel functional imaging—both in vitro and in vivo—with the red emission and high sensitivity of CaRuby-Nano being an ideal match for numerous other indicators emitting in the green-yellow spectral band.

## Discussion

We have developed a high-affinity red-emitting calcium indicator. This novel indicator, CaRuby-Nano, has a K_D_ of 295 nM (for the dextran conjugate). This makes CaRuby-Nano only slightly higher affinity than the commonly used green emitting indicator Fluo-4 (335 nM). On calcium binding CaRuby-Nano shows a 50-fold fluorescence increase (vs. 14-fold and 100-fold for OGB-1 and Fluo-4, respectively). The quantum efficiency of calcium bound CaRuby-Nano (0.45) is lower than that of OGB-1 (∼0.7) but significantly higher than that of Fluo-4 (∼0.14). These values classify CaRuby-Nano as an ideal indicator for the quantification of small intracellular [Ca^2+^] transients (see Appendix 1). Using a range of different experiments we have demonstrated that CaRuby-Nano is well suited for both in vitro and in vivo imaging experiments requiring high sensitivity to [Ca^2+^] changes. Finally, we show that CaRuby-Nano can be combined with activity indicators emitting in the green-yellow spectral band, to allow multiplexed imaging. The versatility of the probe is further increased by the azido function, which can easily be reduced to an amine group, thus opening the field to functionalization with numerous molecular tools such as antibodies, benzylguanine (SNAP tag) or peptides to facilitate specific sub-cellular targeting.

CaRuby-Nano's sensitivity and potential for spectral multiplexing allows sophisticated experiments such as simultaneously measuring pre- and postsynaptic activity or imaging of different signaling modalities in the same cell, allowing previously elusive questions to be directly addressed.

## Materials and methods

### General chemical methods

All the solvents were of analytical grade. Chemicals were purchased from commercial sources. ^1^H-NMR and ^13^C-NMR were measured on a Bruker Avance III-300 MHz spectrometer (Bruker Biospin, The Woodlands, TX, USA) with chemical shifts reported in ppm (TMS as internal standard). Mass spectra were measured on a Focus GC/DSQ II spectrometer (ThermoScientific, Waltham, MA, USA) for IC and an API 3000 spectrometer (Applied Biosystems, PE Sciex) for ES. All pH measurements were made with a Mettler Toledo pH meter. Fluorescence spectra were recorded on a JASCO FP-8300 spectrofluorometer (JASCO, Easton, MD, USA). Absorption spectra were determined on a VARIAN CARY 300 Bio UV-Visible spectrophotometer. All measurements were done at a temperature of 25°C. The purity of the dyes were checked by RP-HPLC C-18, elutant: ACN 0.1% TFA/Water 0.1% TFA, method: 20/80 to 100/0 within 20 min then 100/0 for 10 min detection at λ_Abs_ = 254 nm. The apparent dissociation constant for calcium (K_D_ Ca^2+^) was measured with a calcium calibration buffer kit from Invitrogen (Lifetechnologies, USA). All mass spectra, NMR spectra and chromatograms are included as supplemental data.

### Design of CaRuby-Nano from the first generation CaRubies

In order to develop a high affinity CaRuby, three modifications were carried out based on the first generation CaRubies. First, an oxygen atom was introduced on one of the BAPTA's cycles in order to electronically enrich the latter. Then, this oxygen atom served as an anchor to a spacer terminated by an azide function for further functionalizations either by click chemistry or by reducing it into an amine for coupling with for example, a carboxylic acid. Finally, the fluorophore moiety, an extended rhodamine which is positively charged and therefore has an electron withdrawing effect, was moved from the *para* position of the aniline to the *meta* position. As expected, these modifications lead to a significant increase of affinity towards calcium, yielding a CaRuby with a dissociation constant of 258 ± 8 nM.

### Synthesis of CaRuby-Nano

**Figure 5.**
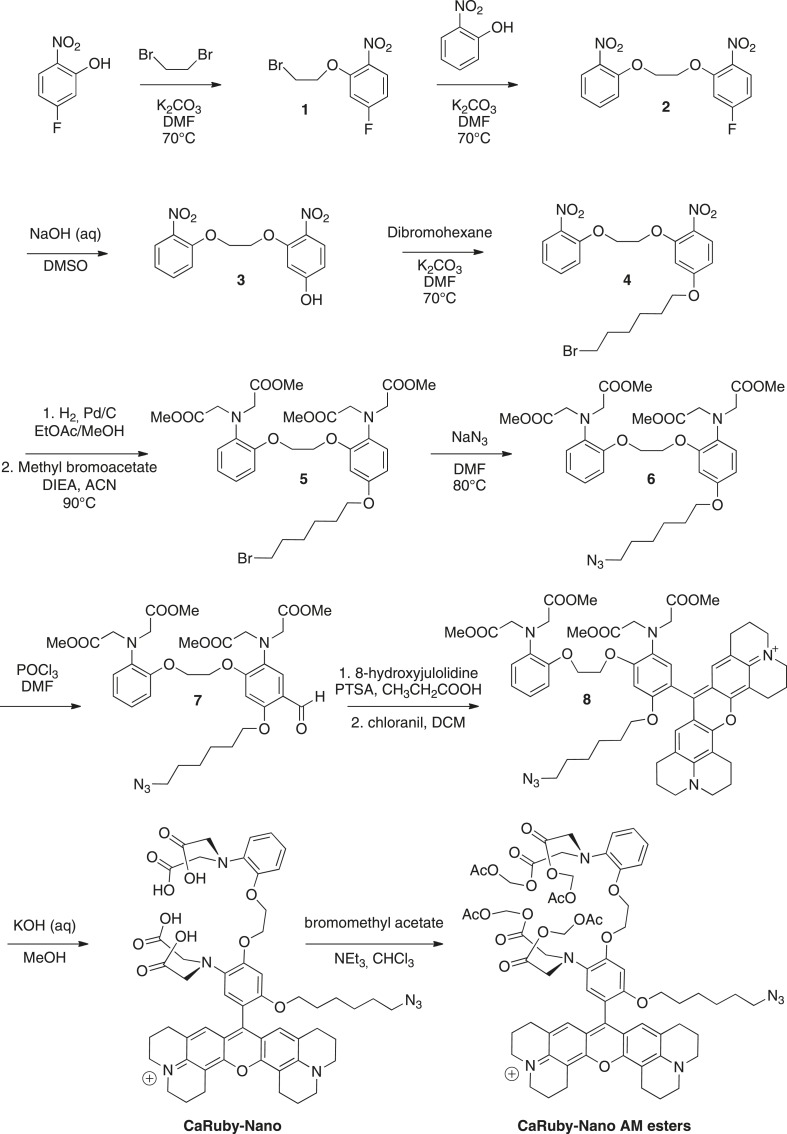
Synthesis of CaRuby-Nano and CaRuby-Nano AM ester. **DOI:** http://dx.doi.org/10.7554/eLife.05808.012

The synthesis pathway is displayed in Figure 5 along with the compound numbering. The NMR and mass spectra for both intermediate compounds and final products are contained in Supplementary file 1.

To a solution of de 5-fluoro-2-nitrophenol (14.90 g, 94.84 mmol) in DMF (75 ml) were added dibromoethane (40.90 ml, 472.2 mmol, 5 eq) and K_2_CO_3_ (26.30 g, 189.7 mmol, 2 eq), the mixture was allowed to stir at 70°C for 2 hr. The solvents were evaporated and the product was extracted with EtOAc washed with water (three times) and brine (two times). The organic phase was dried over MgSO_4_, filtered and evaporated to reach a volume of 200 ml. The symmetric dinitro compound crystallizes first and was filtered off. The filtrate was then allowed to crystallize to obtain 20.12 g of **1** (80%) as a yellow powder. ^*1*^*H-NMR* (300 MHz, DMSO-d6): δ 8.04 (dd, *J*_a-b_ = 9.1 Hz, *J*_a-F_ = 6.1 Hz, 1H, H_a_), 7.37 (dd, *J*_c-F_ = 11.0 Hz, *J*_c-b_ = 2.6 Hz, 1H, H_c_), 7.02 (ddd, *J*_b-a_ = 9.1, *J*_b-F_ = 7.8 Hz, *J*_b-c_ = 2.6 Hz, 1H, H_b_), 4.56–4.53 (m, 2H, CH_2_O), 3.84−3.81 (m, 2H, CH_2_Br). ^*13*^*C-NMR* (75 MHz, DMSO-d6): δ 164.82 (d, ^1^*J*_F-C_ = 251 Hz, CF), 152.81(d, ^3^*J*_C-F_ = 12 Hz, CO), 136.17 (d, ^4^*J*_F-C_ = 3 Hz, CNO_2_), 127.62 (d, ^3^*J*_F-C_ = 11 Hz, C_a_), 108.01 (d, ^2^*J*_F-C_ = 23 Hz, C_b_), 103.45 (d, ^2^*J*_F-C_ = 27 Hz, C_c_), 69.78 (CH_2_O), 30.39 (CH_2_Br). MS (CI), calculated for C_8_H_11_BrFN_2_O_3_ [M + NH_4_]^+^ 280.9, found 281.0.

To a solution of **1** (19.79 g, 74.96 mmol) in DMF (75 ml) were added 2-nitrophenol (11.46 g, 82.45 mmol, 1.1 eq) and K_2_CO_3_ (15.63 g, 112.4 mmol, 1.5 eq), the mixture was allowed to stir overnight at 70°C. The solvent was evaporated and the product was extracted with DCM, washed with HCl (1 M) and brine (2 times). The organic phase was dried over MgSO_4_, filtered and evaporated to reach a volume of 200 ml. The product crystallized and was filtered to obtain 12.00 g of **2** (50%) as a yellow powder. ^*1*^*H-NMR* (300 MHz, DMSO-d6): δ 8.01 (dd, *J*_a-b_ = 9.1 Hz, *J*_a-F_ = 6.1 Hz, 1H, H_a_), 7.86 (dd, *J*_*g-f*_ = 8.1 Hz, *J*_g-e_ = 1.6 Hz, 1H, H_g_), 7.67 (ddd, ^3^*J* = 8.5, 7.4, ^4^*J*_e-g_ = 1.7 Hz, 1H, H_e_), 7.45-7.39 (m, 2H, H_c_, H_d_), 7.15 (ddd, ^3^*J* = 8.1 Hz, 7.4 Hz, ^4^*J* = 1.1 Hz, 1H, H_f_), 7.01 (ddd *J*_b-a_ = 9.1, *J*_b-F_ = 7.8 Hz, *J*_b-c_ = 2.6 Hz, 1H, H_b_), 4.59-4.54 (m, 4H, 2CH_2_O). ^*13*^*C-NMR* (75 MHz, DMSO-d6): δ 164.86 (d, ^1^*J*_F-C_ = 251 Hz, CF), 153.34 (d, ^3^*J*_C-F_ = 11.9 Hz, CO), 150.82 (Cq Ar), 139.74 (Cq Ar), 136.15 (d, ^4^*J*_F-C_ = 3.7 Hz, CNO_2_), 134.33 (C_e_), 127.58 (d, ^3^*J*_F-C_ = 11 Hz, C_a_), 124.85 (C_g_), 121.05 (C_f_), 115.55 (C_d_), 107.90 (d, ^2^*J*_F-C_ = 24 Hz, C_b_), 103.57 (d, ^2^*J*_F-C_ = 27.7 Hz, C_c_), 68.57 (CH_2_O), 67.93 (CH_2_O). MS (ES+), calcd for C_14_H_11_FN_2_O_6_Na [M + Na]^+^ 345.0, found 345.3. HRMS (ES+), calcd for C_14_H_11_FN_2_O_6_Na [M + Na]^+^ 345.0493, found 345.0501.

To a stirred solution of **2** (5.91 g, 18.34 mmol) in DMSO (53 ml) was added NaOH 20% (11.5 ml) the solution turned yellow and was allowed to stir at room temperature overnight. 50 ml of water and 10 ml HCl (1 M) were then added and the product was extracted three times with EtOAc. The organic phase was washed three times with water before being dried over MgSO_4_, the solution was filtered and evaporated and crystallized in EtOAc to obtain 4.46 g of **3** (76%) as a yellow powder. ^*1*^*H-NMR* (300 MHz, DMSO-d6): δ 7.90–7.85 (m, 2H, H_a_, H_g_), 7.67–7.64 (dd, ^3^*J* = 8.7 Hz, ^4^*J* = 1.5 Hz, 1H, H_e_), 7.48 (d, ^3^*J* = 8.4 Hz, 1H, H_d_), 7.16 (t, ^3^*J* = 7.7 Hz, 1H, H_f_), 6.66 (d, ^4^*J* = 2.2 Hz, 1H, H_c_), 6.51 (dd, ^3^*J* = 9.0, ^4^*J* = 2.2 Hz, 1H, H_b_), 4.55–4.54 (m, 2H, CH_2_O), 4.45 (t, *J* = 3.8 Hz, 2H, CH_2_O). ^*13*^*C-NMR* (75 MHz, DMSO-d6): δ 163.87 (Cq Ar), 154.51 (Cq Ar), 150.98 (Cq Ar), 139.79 (Cq Ar), 134.36 (C_e_), 131.12 (Cq Ar), 128.18 (C_a_ or C_g_), 124.86 (C_a_ or C_g_), 121.03 (C_f_), 115.75 (C_d_), 108.01 (C_b_), 101.56 (C_c_), 68.06 (CH_2_O), 67.87 (CH_2_O). MS (CI), calcd for C_14_H_16_N_3_O_7_ [M + NH_4_]^+^ 338.0, found 337.7. HRMS (ES+), calcd for C_14_H_13_N_2_O_7_ [M + H]^+^ 321.0717, found 321.0722.

To a solution of **3** (4.86 g, 15.19 mmol) in DMF (50 ml) were added dibromohexane (11.12 ml, 45.56 mmol, 3 eq) and K_2_CO_3_ (3.16 g, 22.78 mmol, 1.5 eq). The mixture was allowed to stir at 70°C for 12 hr. The solvents were evaporated and the product was extracted with EtOAc washed with water (three times) and brine (two times). The organic phase was dried over MgSO_4_, filtered and evaporated. The crude was purified by column chromatography on silica gel (Cyclohexane/EtOAc: 7/3) to obtain the crude **4** which was crystallized in a mixture of EtOAc and cyclohexane (3/7) to obtain 2.97 g of pure **4** (40%) as a off white powder. Rf = 0.22 (Cyclohexane/EtOAc, 7/3). ^*1*^*H-NMR* (300 MHz, CDCl_3_): δ 8.00 (d, *J* = 9.1 Hz, 1H, H_a_), 7.86 (dd, *J* = 8.1, 1.6 Hz, 1H, H_g_), 7.64–7.58 (m, 1H, H_e_), 7.33 (dd (in solvent pick), 1H, Hd), 7.14–7.09 (m, 1H, H_f_), 6.66 (d, *J* = 2.4 Hz, 1H, H_c_), 6.57 (dd, *J* = 9.1, 2.4 Hz, 1H, H_b_), 4.60–4.51 (m, 4H, 2CH_2_O), 4.08 (t, *J* = 6.4 Hz, 2H, CH_2_O), 3.47 (t, *J* = 6.7 Hz, 2H, CH_2_Br), 1.96–1.85 (m, 4H, 2CH_2_), 1.56 (dt, *J* = 7.1, 3.5 Hz, 4H, 2CH_2_). ^*13*^*C-NMR* (75 MHz, CDCl_3_): δ 164.35 (Cq), 154.63 (Cq), 151.96 (Cq), 140.43 (Cq), 134.37 (C_e_), 133.34 (Cq), 128.32 (C_a_), 125.56 (C_g_), 121.43 (C_f_), 116.14 (C_d_), 106.89(C_b_), 102.02 (C_c_), 68.83 (CH_2_O), 68.70 (CH_2_O), 68.65 (CH_2_O), 33.81 (CH_2_Br), 32.63 (CH_2_), 28.85 (CH_2_), 27.86 (CH_2_), 25.19 (CH_2_). MS (ES+), calcd for C_20_H_23_BrN_2_O_7_Na [M + Na]^+^ 505.0, found 505.5. HRMS (ES+), calcd for C_20_H_24_BrN_2_O_7_ [M + H]^+^ 483.0767, found 483.0772.

To a solution of **4** (5.00 g, 10.35 mmol) in EtOAc (100 ml) and methanol (30 ml) was added Pd/C (1.10 g). The solution was stirred and degassed before H_2_ was allowed to bubble in the solution for 5 hr. The solution was then filtered off celite and rinsed with EtOAc under an atmosphere of argon. The solvents were evaporated and the crude was dissolved in acetonitrile (50 ml), to this solution were added, methyl bromoacetate (12.0 ml, 124.2 mmol, 12 eq) and DIEA (23.0 ml, 124.2 mmol, 12 eq) before being warmed up to 80°C. The solution was allowed to stir overnight at 80°C. The solvents were evaporated, the product was extracted with DCM and washed with water. The organic phase was dried over MgSO_4_, filtered and evaporated. The crude was purified by column chromatography on silica gel (Cyclohexane/EtOAc: 7/3) to obtain 3.71 g of **5** (50%) as a yellowish syrup containing impurities (between 2 and 3 ppm in ^1^H NMR) that could not be removed. Rf = 0.51 (Cyclohexane/EtOAc, 6/4). ^*1*^*H-NMR* (300 MHz, CDCl_3_): δ 6.85–6.74 (m, 5H), 6.39 (d, *J* = 2.7 Hz, 1H), 6.31 (dd, *J* = 8.7, 2.7 Hz, 1H), 4.20 (m, 4H, CH_2_O), 4.08 (s, 4H, 2CH_2_N), 4.02 (s, 4H, 2CH_2_N), 3.81 (t, *J* = 6.4 Hz, 2H, CH_2_O), 3.50 (d, *J* = 7.4 Hz, 12H, 4 OMe), 3.36 (t, *J* = 6.8 Hz, 2H, CH_2_Br), 1.85–1.80 (m, 2H, CH_2_), 1.71-1.67 (m, 2H, CH_2_), 1.42 (t, *J* = 3.6 Hz, 4H, 2 CH_2_). MS (ES+), calcd for C_32_H_43_BrN_2_O_11_Na [M + Na]^+^ 735.2, found 735.8.

To a solution of **5** (3.71 g, 5.218 mmol) in DMF (10 ml) was added NaN_3_ (1.02 g, 15.65 mmol, 3 eq). The solution was stirred at 80°C overnight. The product was extracted with EtOAc and washed with water (three times) and brine (two times), the organic phase was dried over MgSO_4_, filtered and evaporated to give 3.52 g of **6** (quant) as a yellowish syrup. ^*1*^*H-NMR* (300 MHz, CDCl_3_): δ 6.84–6.73 (m, 5H), 6.39 (d, *J* = 2.5 Hz, 1H), 6.31 (dd, *J* = 8.7, 2.5 Hz, 1H), 4.19 (d, 4H, CH_2_O), 4.08 (s, 4H, 2CH_2_N), 4.01 (s, 4H, 2CH_2_N), 3.81 (t, *J* = 6.4 Hz, 2H, CH_2_O), 3.48 (d, *J* = 7.3 Hz, 12H, 4 OMe), 3.20 (t, *J* = 6.8 Hz, 2H, CH_2_N_3_), 1.70–1.64 (m, 2H, CH_2_), 1.60–1.51 (m, 2H, CH_2_), 1.38 (m, 4H, 2 CH_2_). Impurities between 2 and 3 ppm could not be removed. MS (ES+), calcd for C_32_H_44_N_5_O_11_ [M + H]^+^ 674.3, found 674.3. HRMS (ES+), calcd for C_32_H_44_N_5_O_11_ [M + H]^+^ 674.3032, found 674.3054.

To a solution of **6** (1.22 g, 1.81 mmol) in DMF (5 ml) was added POCl_3_ (1.35 ml, 14.48 mmol, 8 eq) dropwise without cooling. After addition the solution was allowed to stir for 40 min and then water (50 ml) was added followed by slow addition of a saturated solution of NaHCO_3_ to reach a pH of 8. The product was extracted with DCM and washed twice with brine before being dried over MgSO_4_ filtrated and evaporated. The crude was purified by column chromatography on silica gel (Cyclohexane/EtOAc: 6/4) to obtain 505 mg of **7** (40%) as a yellow syrup. Rf = 0.25 (Cyclohexane/EtOAc, 5/5). ^*1*^*H-NMR* (300 MHz, CDCl_3_): δ 10.23 (s, 1H, CHO), 7.27 (s, 1H, Ha), 6.86–6.75 (m, 4H, Hd, He, Hf, Hg), 6.39 (s, 1H, Hc), 4.26 (d, *J* = 2.4 Hz, 4H, 2CH_2_O), 4.06 (d, *J* = 3.3 Hz, 4H, 2CH_2_N), 4.02 (d, *J* = 5.8 Hz, 4H, 2CH_2_N), 3.96 (t, *J* = 6.3 Hz, 2H, CH_2_O), 3.49 (2 s, 12H, 2 OMe), 3.22 (t, *J* = 6.8 Hz, 2H, CH_2_N_3_), 1.77 (t, *J* = 7.1 Hz, 2H, CH_2_), 1.57 (t, *J* = 7.0 Hz, 2H, CH_2_), 1.45–1.35 (m, 4H, CH_2_). ^*13*^*C-NMR* (75 MHz, CDCl_3_): δ 187.99 (CHO), 171.94 (COOMe), 171.64 (COOMe), 158.88 (Cq Ar), 157.35 (Cq Ar), 150.21 (Cq Ar), 139.40 (Cq Ar), 133.40 (Cq Ar), 122.44 (CH Ar), 121.86 (CH Ar), 119.19 (CH Ar), 118.28 (Cq Ar), 118.07 (Ca), 113.45 (CH Ar), 97.79 (Cc), 68.93 (CH_2_O), 67.53 (CH_2_O), 66.81 (CH_2_O), 53.36 (2CH_2_N), 53.32 (2CH_2_N), 51.69 (OMe), 51.65 (OMe), 51.35 (CH_2_N_3_), 30.19 (CH_2_), 29.07 (CH_2_), 28.82 (CH_2_), 26.92 (CH_2_), 26.50 (CH_2_), 25.70 (CH_2_). MS (ES+), calcd for C_33_H_44_N_5_O_12_ [M + H]^+^ 702.3, found 702.2. HRMS (ES+), calcd for C_33_H_44_N_5_O_12_ [M + H]^+^ 702.2981, found 702.3008.

The position of the carbonyl was confirmed by further NMR investigations using a Heteronuclear Multiple Bond Correlation (Supplementary file 2).

To a solution of aldehyde **7** (300 mg, 0.428 mmol) in propionic acid (5 ml) was added 8-hydroxyjulolidine (161 mg, 0.856 mmol, 2 eq) and PTSA (8 mg, 0.042 mmol, 0.1 eq). The solution was protected from light and stirred at room temperature overnight. To the brown mixture was added a solution of chloranil (103 mg, 0.428 mmol, 1 eq) in DCM (10 ml), the reaction turned dark and was allowed to stir overnight at room temperature. The dark purple solution was evaporated to dryness. The crude was purified by column chromatography on silica gel (gradient of 100% DCM to 9/1 DCM/Methanol) to obtain 130 mg of **8** (30%) as a purple solid after lyophilisation (dioxane/water: 1/1). Rf = 0.32 (DCM/MeOH, 9/1). ^*1*^*H-NMR* (300 MHz, CDCl_3_): δ 7.84 (d, *J* = 8.1 Hz, 1H, H Ar), 7.06 (d, *J* = 7.9, 1H, H Ar), 6.97–6.86 (m, 5H, H Ar, H_7_), 6.71 (d, *J* = 2.9 Hz, 1H, H Ar), 4.47–4.40 (m, 4H, CH_2_O), 4.21 (s, 4H, NCH_2_COOMe), 4.11 (s, 4H, NCH_2_COOMe), 3.87 (t, *J* = 6.1 Hz, 2H, CH_2_O), 3.67 (s, 6H, 2 OMe), 3.56 (m, 14H, 2 OMe, H_1_, H_4_), 3.11 (d, *J* = 7.0 Hz, 2H, CH_2_N_3_), 3.04 (t, *J* = 6.3 Hz, 4H, H_6_), 2.75 (q, *J* = 6.2 Hz, 4H, H_3_), 2.13–2.10 (m, 4H, H_5_), 2.00 (t, *J* = 5.5 Hz, 4H, H_2_), 1.49–1.34 (m, 4H, CH_2_), 1.19–1.03 (m, 4H, CH_2_). ^*13*^*C-NMR* (75 MHz, CDCl_3_): δ 171.97 (CO ester), 171.56 (CO ester), 153.04 (C Ar), 152.74 (C Ar), 152.31 (C Ar), 152.09 (C Ar), 151.02 (C Ar), 150.43 (C Ar), 144.79 (C Ar), 139.41 (C Ar), 138.16 (C Ar), 132.61 (C Ar), 128.20 (CH Ar), 127.15 (CH Ar), 126.33 (CH Ar), 123.34 (C Ar), 122.64 (CH Ar), 122.61 (CH Ar), 121.91 (CH Ar), 119.54 (CH Ar), 113.89 (C Ar) (CH Ar), 113.43 (C Ar), 113.35 (C Ar), 105.16 (C Ar), 69.10 (CH_2_O), 67.70 (CH_2_O), 67.19 (CH_2_O), 53.66 (NCH_2_COOMe), 53.52 (NCH_2_COOMe), 51.73 (4 OMe), 51.16 (CH_2_N_3_), 50.97 (C_1_ or C_4_), 50.52 (C_1_ or C_4_), 28.82 (CH_2_), 28.73 (CH_2_), 27.72 (C_3_), 26.26 (CH_2_), 25.52 (CH_2_), 20.83 (C_2_), 20.00 (C_6_), 19.85 (C_5_). MS (ES+), calcd for C_57_H_68_N_7_O_12_ [M]^+^ 1042.5, found 1042.9. HRMS (ES+), calcd for C_57_H_68_N_7_O_12_ [M]^+^ 1042.4920, found 1042.4949.

To a solution of **8** (100 mg, 0.090 mmol) in methanol (6 ml) were added, KOH (504 mg, 9.00 mmol, 100 eq) followed by 2 ml of water, the mixture was stirred overnight. The product was washed with HCl (1 M) and extracted with CHCl_3_ until the aqueous phase become slightly pink. The organic phase was then dried over MgSO_4_, filtered and evaporated. The crude was purified on a reverse phase column C-18 using acetonitrile (0.1% TFA) and water (0.1% TFA) as eluant (20% ACN to 60%). The solvents were evaporated and 80 mg of *CaRuby-Nano* (∼90%) were obtained as a purple solid after lyophilisation (dioxane/water, 1/1). MS (ES+), calcd for C_53_H_60_N_7_O_12_ [M]^+^ 986.4, found 986.4. HRMS (ES+), calcd for C_53_H_60_N_7_O_12_ [M]^+^ 986.4294, found 1042.4329.

To a solution of *CaRuby-Nano* (50 mg, ∼50 µmol) in chloroform were added bromomethyl acetate (80 µl, 500 µmol, 1 eq) and NEt_3_ (60 µl, 400 µmol, 8 eq). The solution was protected from light and allowed to stir at room temperature overnight. The reaction was monitored by TLC (DCM/MeOH, 9/1). The solvents were evaporated and the crude was purified by column chromatography on silica gel (gradient of 100% DCM to 9/1 DCM/Methanol) to obtain 30 mg of *CaRuby-Nano AM esters* (∼45%) as a purple solid after lyophilisation (dioxane/water, 1/1). Rf = 0.45 (DCM/MeOH, 9/1). MS (ES+), calcd for C_65_H_76_N_7_O_20_ [M]^+^ 1274.5, found 1274.5. HRMS (ES+), calcd for C_65_H_76_N_7_O_20_ [M]^+^ 1274.5140, found 1274.5128.

### Synthesis of dextran conjugates

Dextran 6000 MW (Sigma–Aldrich, ref: 31388) and dextran 1500 MW (Sigma–Aldrich, ref: 31394) were propargylated as described by Nielsen et al. (2010). The ^1^H-NMR showed that the functionalized dextrans were propargylated once by unit.

Final MW Dextran 6000: ∼9800 g.mol^−1^.

Final MW Dextran 1500 : ∼2400 g.mol^−1^.

#### Conjugation of dextran 6000

To a solution of propargylated dextran 6000 (30 mg, ∼3 µmol) in water (3 ml) was added **9** (8 mg, 8 µmol, 2.6 eq) in methanol (1 ml) and an heterogeneous solution of CuSO_4_·5H_2_O (4 mg, 16 µmol, 5.3 eq) and sodium ascorbate (4 mg, 20 µmol, 6.6 eq) in water (500 µl). The solution was allowed to stir in the dark at room temperature overnight. The solvents were evaporated and the crude was dissolved in 1 ml of EDTA solution (0.1 M) and passed through a G-25 column to give 24 mg of CaRu-Dextran 6000 conjugate (∼60% yield).

#### Conjugation of dextran 1500

To a solution of propargylated dextran 1500 (30 mg, ∼12.5 µmol) in DMF (1 ml) was added **9** (4.5 mg, 4.5 µmol, 0.3 eq) in DMF (200 µl) and a heterogeneous solution of CuSO_4_·5H_2_O (4 mg, 16 µmol, 1.3 eq) and sodium ascorbate (4 mg, 20 µmol, 1.6 eq) in water (100 µl). The solution was allowed to stir in the dark at 50°C overnight. The solvents were evaporated and the crude was dissolved in 1 ml of EDTA solution (0.1 M) and passed through a G-25 column to give 20 mg of CaRu-Dextran 1500 conjugate (∼58% yield).

### Animals

All procedures were approved by the local ethical review committee and performed under license from the UK Home Office in accordance with the Animals (Scientific Procedures) Act 1986, and in accordance with the Institut National de la Santé et de la Recherche Médicale (INSERM) Animal Care and Use Committee Guidelines and with Centre National de la Recherche Scientifique (CNRS) animal experimentation guidelines and European laws and policies, as applicable.

### Slicing

Parasagittal cerebellar slices (200 μm) were made using standard techniques (Davie et al., 2006) from C57BL6/J mice (Harlan, UK) at postnatal days 25–29. Artificial CSF (ACSF) for both slicing and recording contained the following (in mM): 125 NaCl, 2.5 KCl, 26 NaHCO_3_, 1.25 NaH_2_PO_4_, 25 glucose, 1 MgCl_2_, and 2 CaCl_2_, and was bubbled with 5% carbon dioxide, 95% oxygen. Slices were continuously superfused with ACSF during the experiment.

For high speed imaging experiments, acute 260 µm thick slices were obtained from the cerebellar vermis of P60 CD1 mice and superfused with ACSF, as previously described (Dugue et al., 2009).

### Electrophysiology and imaging in cerebellum and neocortex

Full frame and linescan two-photon imaging was performed using microscopes optimized for in vitro (Prairie Technologies, now Bruker Nano Surfaces, USA) or in vivo (MOM, Sutter, Novato, CA, USA) experiments. Two photon excitation was provided by a pulsed Ti:Sa laser (MaiTai HP, Spectra-Physics, Santa Clara, CA, USA), tuned to a central wavelength of 890–920 nm. The microscopes were controlled by ScanImage 3.5 and 3.7.1 (Pologruto et al., 2003) (now Vidrio Technologies, Arlington, VA, USA).

For two-color imaging of iGluSnFR and CaRuby-Nano the Ti:Sa was tuned to 900 nm. Fluorescence light was split into red and green channels using dichroic mirrors (575/DCXR, Chroma, Bellows Falls, VT, USA) and bandpass filtered (green: 525/50; red: 607/45; both: Semrock, Lake Forest, IL, USA).

Patch-clamp pipettes were filled with an internal solution containing (in mM): K-methanesulfonate 133, KCl 7, HEPES 10, Mg-ATP 2, Na_2_ATP 2, Na_2_GTP 0.5, EGTA 0.05, 0.1 Alexa Fluor 488 and CaRuby-Nano dextran as indicated; pH 7.2. Recordings from visually identified Purkinje cells were made using a Multiclamp 700B amplifier (Molecular Devices, Sunny Vale, CA, USA). Data were lowpass filtered at 4 kHz and acquired at 20 kHz using an ITC-18 digitizer (HEKA Intruments, Bellmore, NY, USA) controlled by AxoGraph X (AxoGraph Scientific, Sydney, Australia). Electrical stimuli were delivered via a theta-glass bipolar electrode filled with ACSF using a constant current stimulus isolator (DS-3, Digitimer, Letchworth Garden City, UK). When using electrical stimulation, 10 µM SR-95531 (Sigma or Tocris) was added to the perfusion medium.

Climbing fiber stimulation-evoked transient [Ca^2+^] changes in Purkinje cell spines were recorded at high acquisition rate (>2 kHz) by two-photon random-access microscopy, a technique based on the use of acousto-optic deflectors (AODs), which enable selective scanning of defined points (Otsu et al., 2008). Purkinje cells were recorded in current-clamp mode, using 2–3 MΩ patch pipettes containing 300 μM CaRuby-Nano dextran. Recordings were obtained by use of a Multiclamp 700B (Molecular Devices). Following the dialysis of CaRuby-Nano, Purkinje cells in slices were imaged under a 25× Leica water immersion objective (HCX IRAPO L 25×/0.95, Leica Microsystems, Wetzlar, Germany). Two-photon excitation was produced by a pulsed Ti:Sa laser (Chameleon Vision Plus, Coherent, Santa Clara, CA, USA) coupled into the transmitted light pathway of the microscope by a dichroic filter (740dcsx, Chroma) and tuned to a central wavelength of 890 nm. A custom-made user interface based on National Instrument cards programmed under Labview (both National Instruments, Austin, TX, USA) was used to operate the AODs and coordinate the scanning protocols and signal acquisition. A multifunction card (NI-PCI-MIO 16 E-4) was used to pass all the triggers necessary to synchronize the imaging and the electrophysiology and to control the piezo-electric device that moves the objective in Z. Fluorescence photons were detected by cooled AsGaP photomultipliers (H7421-40, Hamamatsu, Hamamatsu, Japan) discriminated and counted on a fast digital card.

### Virus injection

Young (P19) C57BL6/J mice were anesthetized using isoflurane, an incision was made into the scalp and a small (∼0.5 mm) craniotomy was performed over lobule V of the cerebellar vermis. A wide bore (∼50 µm) micropipette containing viral suspension (AAV1.hSyn.iGluSnFr.WPRE.SV40, University of Pennsylvania Vector Core) was inserted through the craniotomy and carefully lowered 1.0 mm into the brain. Using application of low pressure 400–800 nl viral suspension were slowly injected (10–20 min). After the injection further 5–10 min were waited before retraction of the injection pipette. The scalp was glued and sutured and the mouse left to recover. At least 7 days incubation time were allowed prior to further experiments.

### In vivo imaging of olfactory sensory neuron terminals

Kv3.1-eYFP mice (Metzger et al., 2002) (8–10 week-old) were anesthetized with an intraperitoneal injection of ketamine (100 mg/kg) and xylazine (10 mg/kg). CaRuby-Nano dextran (6 kDa) was dissolved 2.5% wt/vol in a solution of aCSF (in mM: 125 NaCl, 2.5 KCl, 1.25 NaH_2_PO_4_, 25 NaHCO_3_, 1 MgCl_2_, 2 CaCl_2_ and 25 glucose) with 0.2% Triton X-100 (Sigma–Aldrich). 8 µl of this solution was injected in the mouse naris, and mice were left on their backs to recover from anesthesia (protocol adapted from [Wachowiak and Cohen, 2001; Lecoq et al., 2009]). 7 days later, an acute craniotomy was performed over the dorsal olfactory bulb and the brain stabilized with 3.5% agar for imaging. To activate olfactory sensory neurons (OSNs), odors were applied in a 1 ml/min flux of filtered, humidified air supplemented with 30% oxygen. eYFP and CaRuby-Nano fluorescence was collected in two separate channels (‘green’ and ‘red’, respectively) of a custom-built two-photon laser scanning microscope, with the femtosecond pulsed excitation beam set to 910 nm.

### In vivo bulk loading and imaging

Adult C57BL6 mice (6–9 weeks) were anesthetized with isoflurane, supplemented with 1 mg/kg chlorprothixene. A 1.5–2 mm craniotomy was performed over cerebellar lobule V. Care was taken to leave the dura mater intact. CaRuby-Nano-AM was prepared and injected using standard methods (Stosiek et al., 2003; Sullivan et al., 2005). A 50 µg aliquot was dissolved in 20% Pluronic-127 in DMSO (Invitrogen) and then diluted 1:10 in saline (150 mM NaCl, 2.5 mM KCl, 10 mM HEPES, pH 7.4). This solution was filtered and injected into the cerebellum under visual guidance using a patch-pipette and 500–750 mbar pressure for 1–3 min. After injection the preparation was left to incubate for up to 1.5 hr prior to imaging. This helped improve labeling and lower unspecific fluorescence.

### Data analysis and statistics

Imaging data were analyzed using ImageJ (http://rsbweb.nih.gov/ij/). Extracted fluorescence traces, linescans and electrophysiological data were analyzed using in house routines programmed in Igor Pro versions 5 or 6.2 (Wavemetrics) and in pClamp 10 (Molecular Devices). Statistical analysis was performed in Matlab (MathWorks, Natick, MA, USA) or Igor Pro (Wavemetrics, Portland, OR, USA). Experimental groups were compared using a t-test and were assumed to be significantly different if the found p-values were <0.05.

## Figures and Tables

**Appendix 1 Figure 1.**
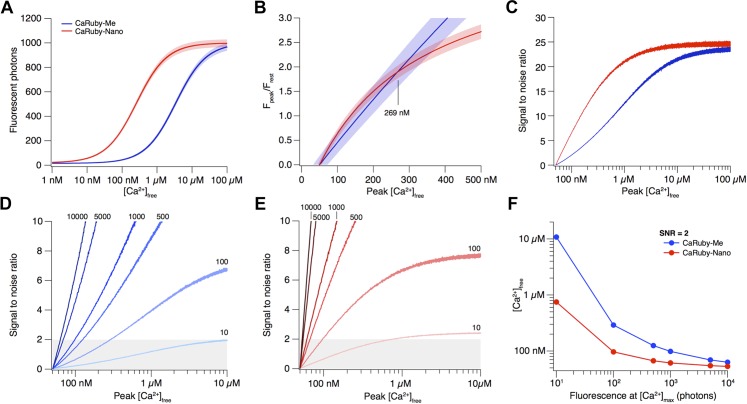
Signal amplitude and SNR depend on indicator K_D_. (**A**) [Ca^2+^]-dependent fluorescence for CaRuby-Me (blue) and CaRuby-Nano (red). The theoretical noise levels (1 SD) are indicated by the translucent areas. (**B**) [Ca^2+^]-dependent dF/F_0_ for both indicators (same colors as in a) with the uncertainties indicated by the areas surrounding the curves. (**C**) [Ca^2+^]-dependent SNR (same color code as in **A**). Note that the noise on the curves is a consequence of the Monte-Carlo simulations. (**D**) SNR curves for CaRuby-Me at different maximum fluorescence levels (maximal photon numbers indicated next to the corresponding curves). The grey area marks the area with SNR < 2. (**E**) SNR curves for CaRuby-Nano at the same brightness levels as in d. (**F**) [Ca^2+^]_peak_ required to reach SNR = 2 for different maximal brightness levels. **DOI:** http://dx.doi.org/10.7554/eLife.05808.015

## Additional files

Supplementary file 1. Spectra (NMR and mass). **DOI:** http://dx.doi.org/10.7554/eLife.05808.013

Supplementary file 2. HMBC data. **DOI:** http://dx.doi.org/10.7554/eLife.05808.014

## Appendix 1

Technical Background: signal amplitude and SNR depend on K_D_ The expected dF/F_0_ signal amplitude and signal-to-noise ratio (SNR) of a transient analyte-dependent fluorescence increase is a major factor in determining the suitability of an indicator dye for a given measurement. In Ca^2+^ imaging, the interplay between fluorescence gain (F_max_ = F_peak_/F_resting_), indicator brightness, and indicator K_D_ will determine the signal amplitude and SNR for a given [Ca^2+^] transient amplitude. Assuming that dyes based on the same fluorophore have similar brightness, the K_D_ and the fluorescence gain will determine the suitability of indicators for detecting [Ca^2+^] transients of a given size. Other factors, such as the need to remain in a linear regime of the [Ca^2+^]-to-fluorescence relation or the need to avoid excessive Ca^2+^ buffering, will also play a role in choosing the best suited indicator. As these factors have been discussed in detail previously (Yasuda et al., 2004; Higley and Sabatini, 2008), we here focus only on signal amplitude and SNR. Depending on the size of a [Ca^2+^] transient, different affinity indicators will give the best possible signal and SNR. Here we compare the recently published CaRuby-Me (K_D_ = 3.4 µM; gain = 62.5) and the novel indicator described in this publication, CaRuby-Nano, (K_D_ = 258 nM; gain = 50; see main text for details). The basis for our calculations is following equation describing the relationship between [Ca^2+^] and fluorescence intensity:

$$F_{\left(\left[{\text{Ca}}^{2+}\right]\right)}=\frac{\left[{\text{Ca}}^{2+}\right]}{K_{D}}*F_{\max}+\left(\frac{F_{\max}}{dF}\right)$$

The resulting fluorescence vs [Ca^2+^] curves for both CaRuby-Me and CaRuby-Nano are shown in Appendix figure 1A. The signal size resulting from a given [Ca^2+^] transient is commonly reported as the dF/F_0_ amplitude (Yasuda et al., 2004). Assuming a resting [Ca^2+^] of 50 nM, the [Ca^2+^]_peak_ dependence of dF/F_0_ for CaRuby-Me and CaRuby-Nano is shown in Appendix figure 1B. This analysis suggests that CaRuby-Nano would have a small advantage in signal size over CaRuby-Me for [Ca^2+^] transients not exceeding 269 nM. While this normalized signal size (i.e., fractional change in fluorescence) is the commonly reported value in quantitative microscopy, the signal-to-noise ratio of this signal determines how reliably it can be measured. To analyse the effect of indicator Ca^2+^-affinity on the SNR, we make a number of assumptions:

1. The resting [Ca^2+^] is 50 nM.
2. The brightness of both indicators at maximal [Ca^2+^] is equal.
3. The detection system does not add additional noise sources.
4. The baseline fluorescence is calculated as the average of 50 time points.

As fluorescence is a stochastic quantum event, the measured intensity (i.e., number of detected photons) will be determined by Poisson statistics (Pawley, 2006). In consequence, the noise level of fluorescence is equal to the square root of the fluorescence intensity. To estimate how this noise determines the uncertainty of the calculated dF/F_0_ values and ultimately which SNR can be achieved at a given maximum brightness, we performed a Monte-Carlo analysis: Random Poisson noise was added to the [Ca^2+^]-dependent fluorescence curves shown in Appendix figure 1A, this was repeated 10,000 times. In addition a signal baseline consisting of 50 points was generated by drawing samples from a Poisson distribution with a mean of the fluorescence expected at resting [Ca^2+^]. For each iteration the dF/F_0_ ratio was calculated. Finally the average dF/F_0_ curve as well as the corresponding standard deviation (SD) over all iterations were determined. As the brightness of the sample (i.e., fluorescence intensity at maximal [Ca^2+^]) strongly influences the SNR, this analysis was initially performed at a fixed maximum brightness of 1000 photons (e.g., the signal resulting from four detected fluorescence photons per excitation pulse at a 3 µs pixel dwell time). While the resulting noise appears negligible against the raw Ca^2+^ binding curve in Appendix figure 1A, the propagated error following dF/F_0_ normalization is substantial (Appendix figure 1B) and the resulting [Ca^2+^]-dependent SNR (SNR_([Ca2+])_ = Fluorescence_([Ca2+])_/SD_([Ca2+])_) curves clearly show that as a consequence of the brighter baseline fluorescence the higher affinity CaRuby-Nano is expected to always yield a better SNR than CaRuby-Me under the simulated conditions. Under sufficiently bright conditions, such as those assumed in Appendix figure 1A–C, the much larger signal achievable at [Ca^2+^] over ∼400 nM outweighs this SNR advantage. To see how the balance between SNR and signal amplitude change with changing signal brightness, we determined the SNR curves for varying levels of maximum brightness (from 10 to 10,000 photons; Appendix figure 1D,E). As expected we find that for a given indicator, the dimmer the imaging conditions, the larger a [Ca^2+^] transient needs to be for reliable detection (assumed to require SNR ≥ 2). On the other hand, the required [Ca^2+^] transient amplitude for reliable detection at a given brightness strongly depends on the Ca^2+^ affinity of the indicator, with higher affinity CaRuby-Nano requiring substantially smaller transients than CaRuby-Me (Appendix figure 1F). What is a level of brightness that can be expected in biological imaging? Tan et al. reported a photon-flux range of 1–4 photons per µs at rest when using Oregon–Green 488 BAPTA-1 (Tan et al., 1999) (OGB-1), which would correspond to approximately 3–12 photons at fully saturating [Ca^2+^]. Thus, even when summing the signal from dozens of pixels, maximum brightness is expected to be in the range of 100–500 photons for standard pixel dwell times (1–4 µs). At this brightness CaRuby-Me would require Ca^2+^ transients reaching ∼290 nM [Ca^2+^] to cross the SNR = 2 threshold (just below the level of the transients evoked by a single action potential in CA1 pyramidal neurons [Maravall et al., 2000]) whereas CaRuby-Nano would require transients under 100 nM. Taken together these results show that while under bright conditions low-affinity indicators have the advantage of larger signal amplitudes, under the dimmer conditions often found in biological imaging, lower affinity dyes will often be working at the edge of the detectability. Thus, for quantification of small, localized (i.e., where it is not possible to average many pixels) Ca^2+^ signals, high affinity indicators such as OGB-1 and CaRuby-Nano are needed.
