# Supplementary material for: CaRuby-Nano: a novel high affinity calcium probe for dual color imaging
Source: eLife. 2015 Mar 31;4:e05808. doi: 10.7554/eLife.05808 (PMC4379494; doi:10.7554/eLife.05808)
Supplement: Supplementary file 1. — Spectra (NMR and mass). DOI: http://dx.doi.org/10.7554/eLife.05808.013 [file elife05808s001.zip › spectra/HRMS_CaRubyAM.pdf]

Elemental Composition Report

Single Mass Analysis

Tolerance = 5.0 PPM / DBE: min = -1.5, max = 100.0
Element prediction: Off
Number of isotope peaks used for i-FIT = 9

Monoisotopic Mass, Even Electron Ions
128 formula(e) evaluated with 2 results within limits (all results (up to 1000) for each mass)
Elements Used:
C: 0-100 H: 0-150 N: 7-7 O: 15-25

23-Nov-2012 2::5::7 MeOH+CH2Cl2 LCT Premier XE KE483
ENS_MC526A 45 (1.112) Cm (43:49) 1: TOF MS ES+
1.26e+003

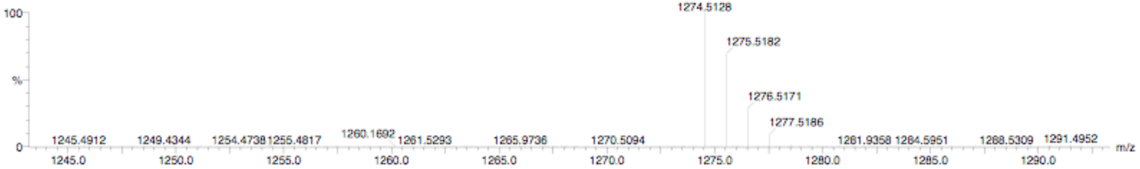

Mass	Calc. Mass	mDa	PPM	DBE	i-FIT	i-FIT (Norm)	Formula
Minimum:				-1.5			
Maximum:		5.0	5.0	100.0			
1274.5128	1274.5145	-1.7	-1.3	31.5	156.9	0.0	C65 H76 N7 O20
	1274.5086	4.2	3.3	40.5	161.6	4.6	C72 H72 N7 O15

HRMS Spectra of **CaRuby-Nano AM esters**
