# Supplementary material for: CaRuby-Nano: a novel high affinity calcium probe for dual color imaging
Source: eLife. 2015 Mar 31;4:e05808. doi: 10.7554/eLife.05808 (PMC4379494; doi:10.7554/eLife.05808)
Supplement: Supplementary file 1. — Spectra (NMR and mass). DOI: http://dx.doi.org/10.7554/eLife.05808.013 [file elife05808s001.zip › spectra/HRMS_Comp3.pdf]

Elemental Composition Report

Single Mass Analysis

Tolerance = 5.0 PPM / DBE: min = -1.5, max = 100.0
Element prediction: Off
Number of isotope peaks used for i-FIT = 9

Monoisotopic Mass, Even Electron Ions
246 formula(e) evaluated with 1 results within limits (all results (up to 1000) for each mass)
Elements Used:
C: 0-100 H: 0-150 N: 0-5 O: 0-10

23-Nov-2012 3::6::6 MeOH+CH2Cl2 LCT Premier XE KE483
ENS_AB008 21 (0.573) Cm (17:31) 1: TOF MS ES+
3.12e+004

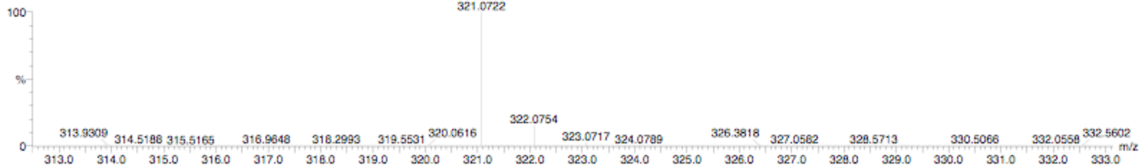

Mass	Calc. Mass	mDa	PPM	DBE	i-FIT	i-FIT (Norm)	Formula
Minimum:				-1.5			
Maximum:		5.0	5.0	100.0			
321.0722	321.0723	-0.1	-0.3	9.5	933.2	0.0	C14 H13 N2 O7

HRMS Spectra of **3**
