# Supplementary material for: CaRuby-Nano: a novel high affinity calcium probe for dual color imaging
Source: eLife. 2015 Mar 31;4:e05808. doi: 10.7554/eLife.05808 (PMC4379494; doi:10.7554/eLife.05808)
Supplement: Supplementary file 1. — Spectra (NMR and mass). DOI: http://dx.doi.org/10.7554/eLife.05808.013 [file elife05808s001.zip › spectra/MS_Comp7.pdf]

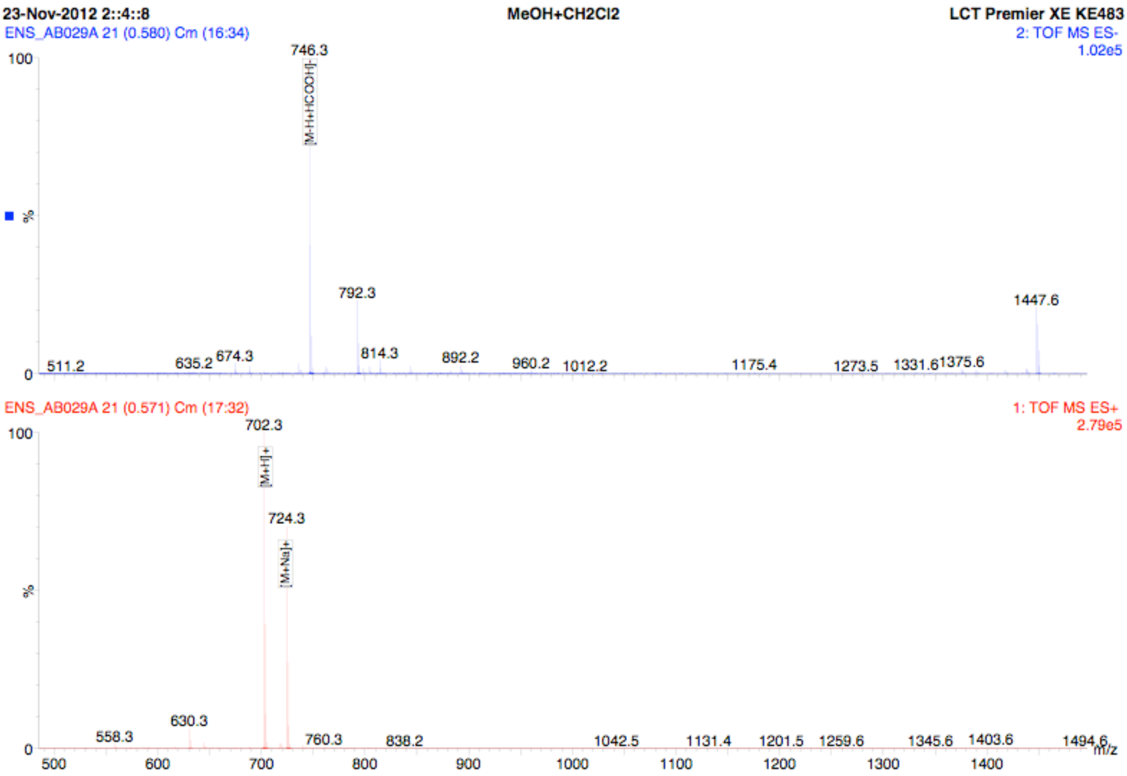

MS Spectra of **7**
