# Supplementary figures and images for: CaRuby-Nano: a novel high affinity calcium probe for dual color imaging
Source: eLife. 2015 Mar 31;4:e05808. doi: 10.7554/eLife.05808 (PMC4379494; doi:10.7554/eLife.05808)

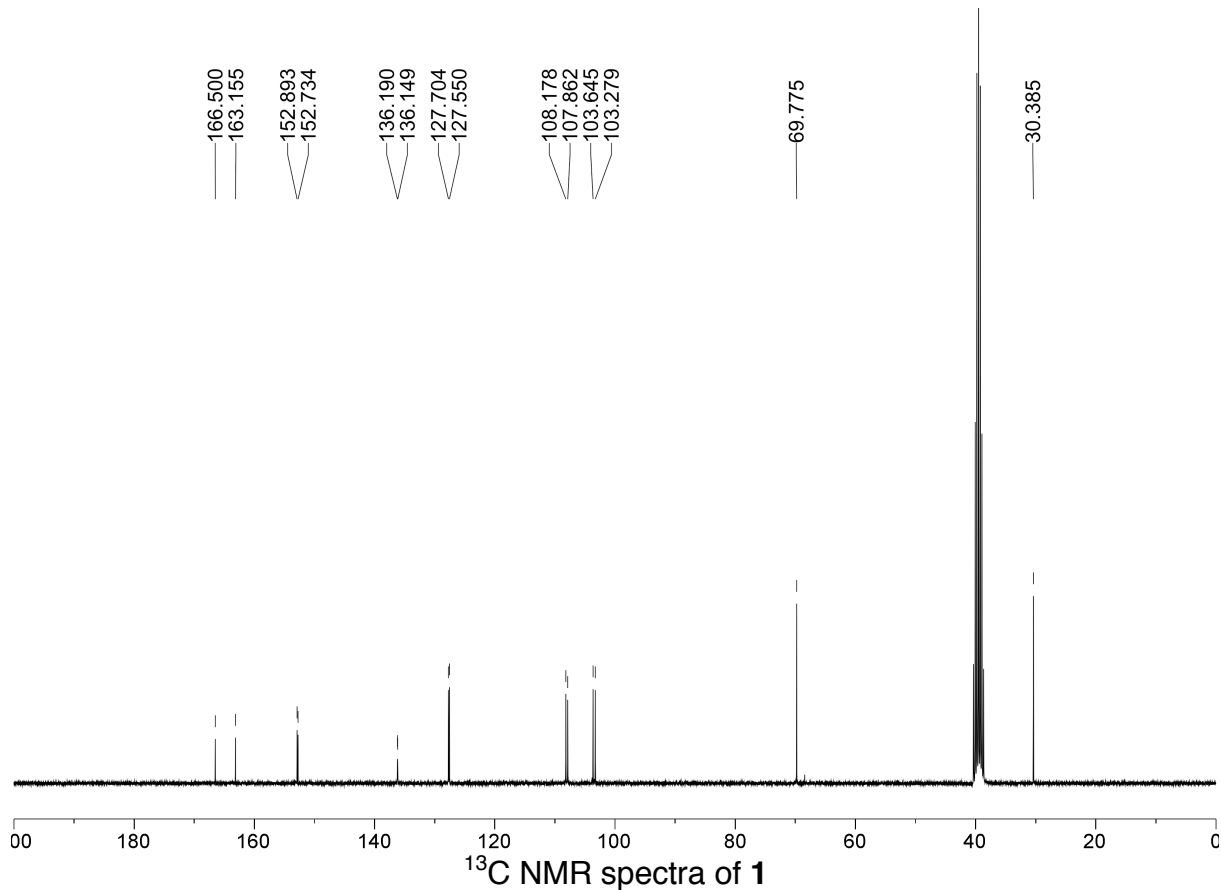

Supplement: Supplementary file 1. — Spectra (NMR and mass). DOI: http://dx.doi.org/10.7554/eLife.05808.013 [file elife05808s001.zip › spectra/C-NMR_Comp1.pdf]

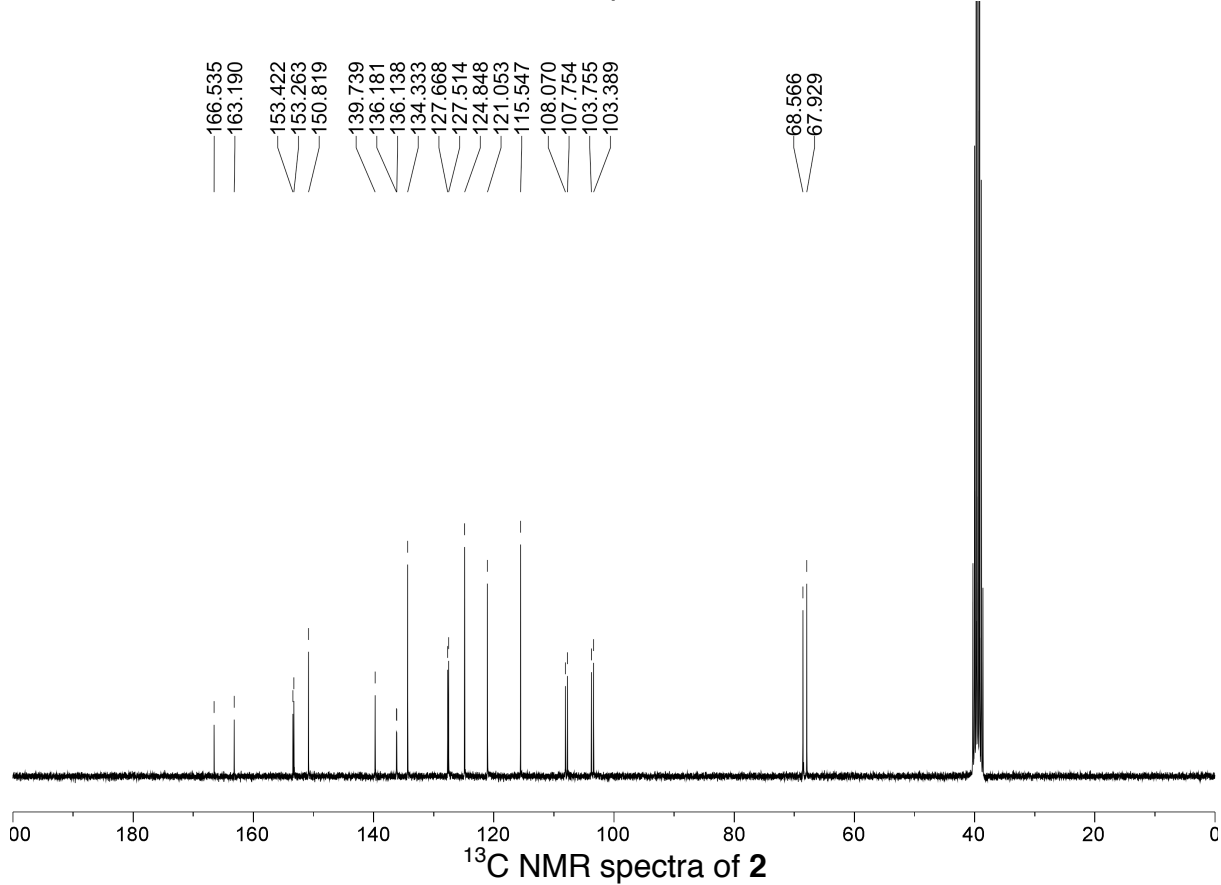

Supplement: Supplementary file 1. — Spectra (NMR and mass). DOI: http://dx.doi.org/10.7554/eLife.05808.013 [file elife05808s001.zip › spectra/C-NMR_Comp2.pdf]

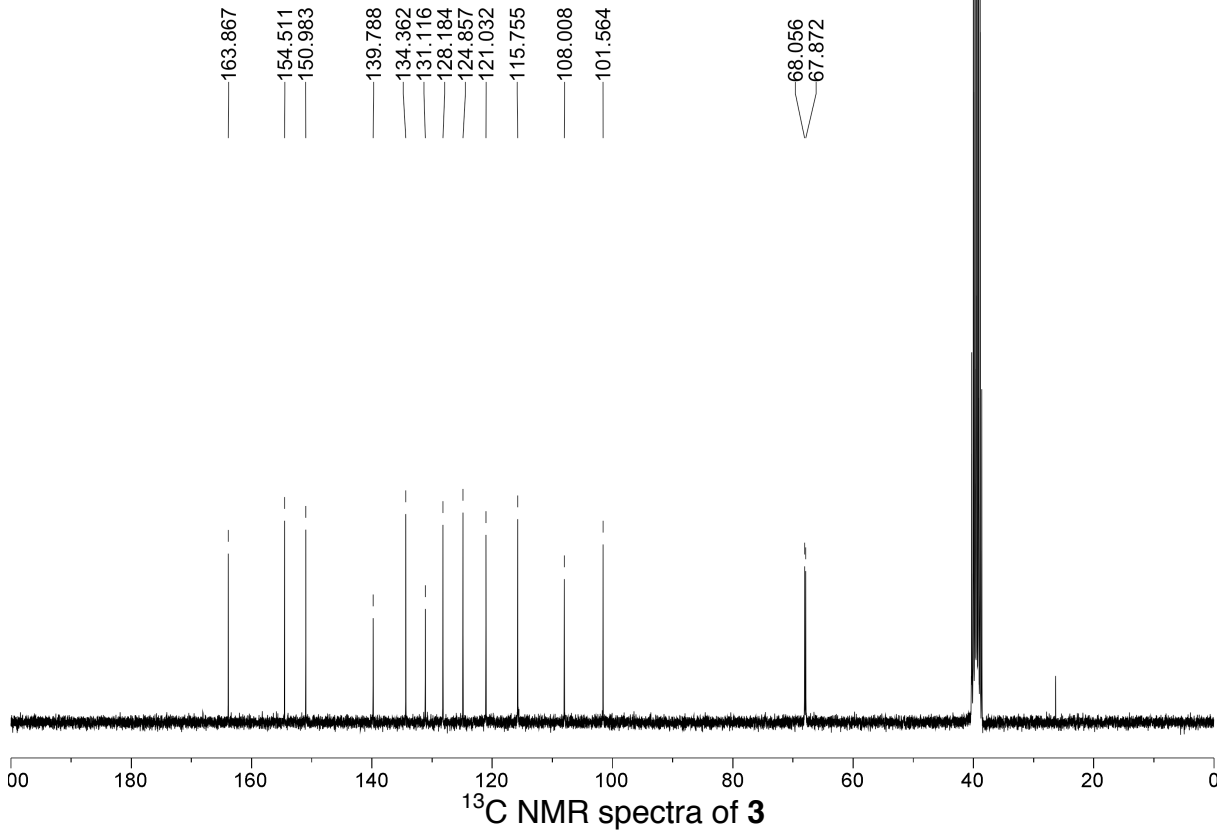

Supplement: Supplementary file 1. — Spectra (NMR and mass). DOI: http://dx.doi.org/10.7554/eLife.05808.013 [file elife05808s001.zip › spectra/C-NMR_Comp3.pdf]

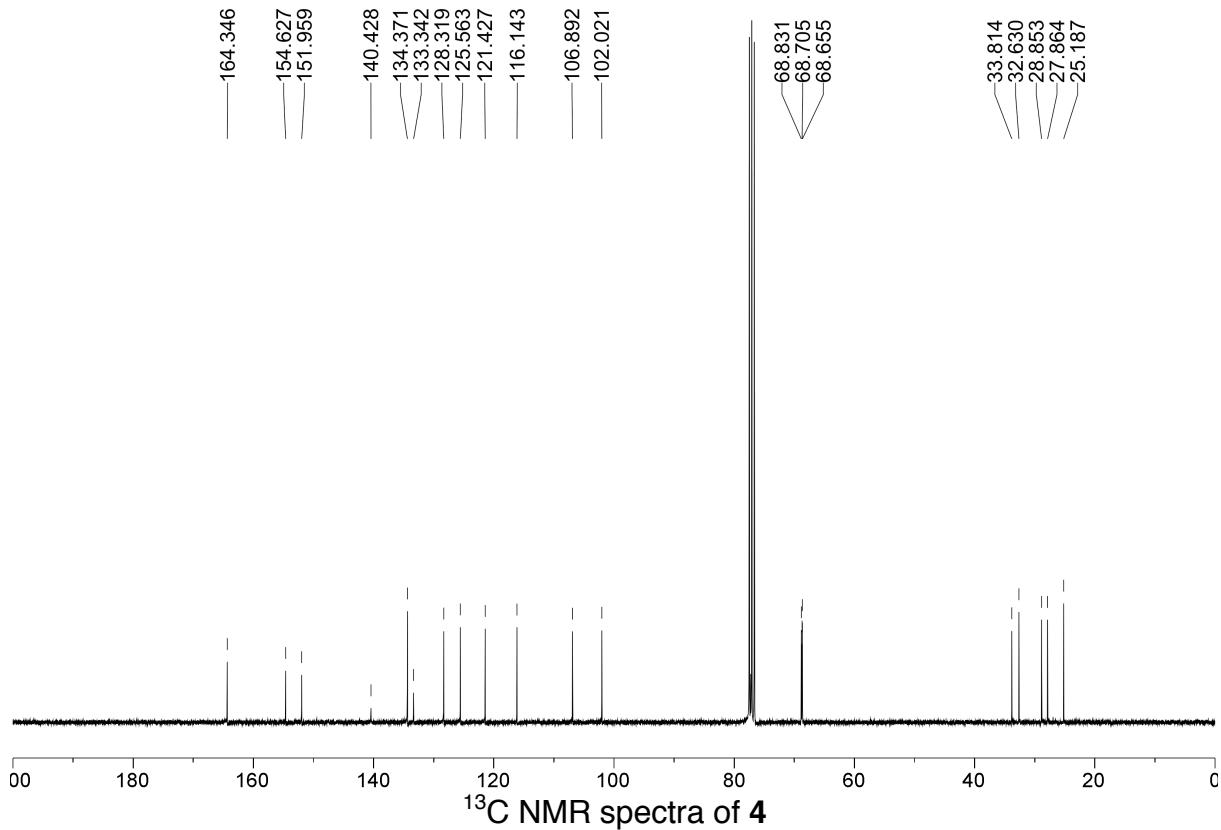

Supplement: Supplementary file 1. — Spectra (NMR and mass). DOI: http://dx.doi.org/10.7554/eLife.05808.013 [file elife05808s001.zip › spectra/C-NMR_Comp4.pdf]

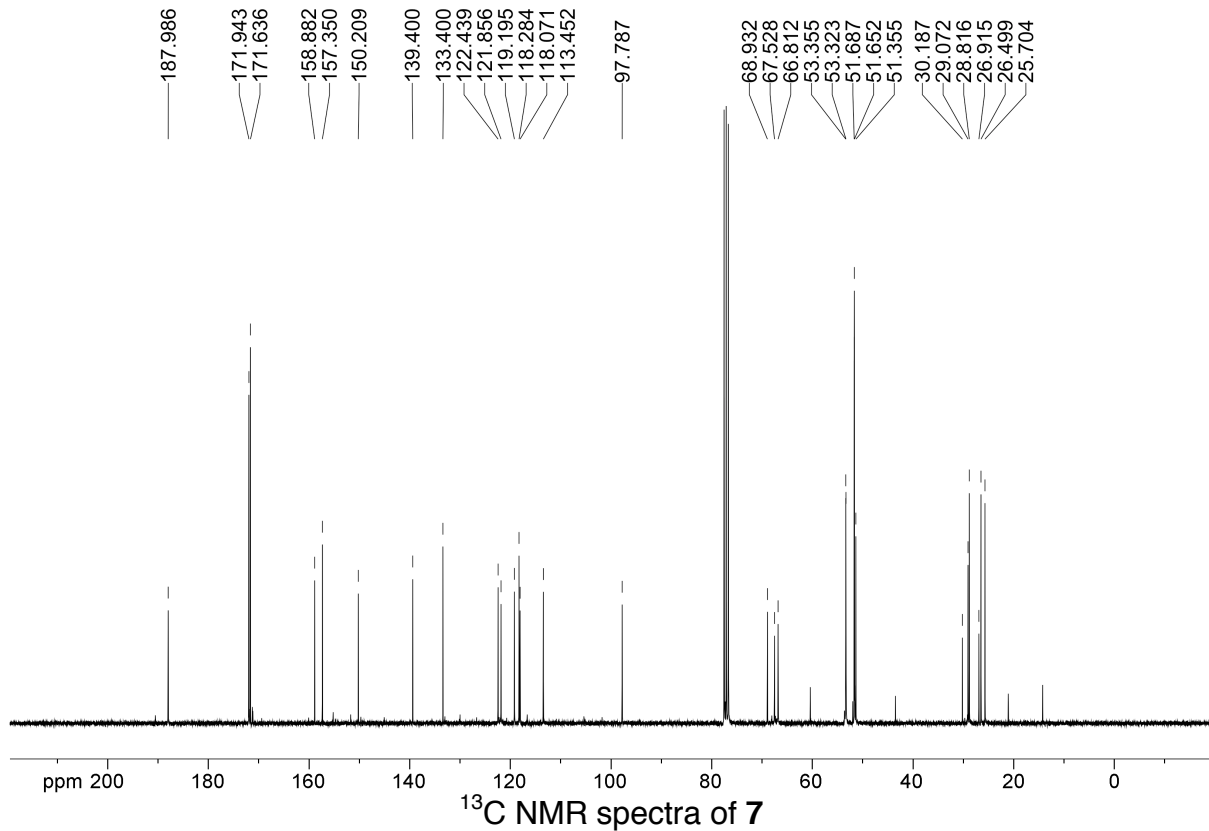

Supplement: Supplementary file 1. — Spectra (NMR and mass). DOI: http://dx.doi.org/10.7554/eLife.05808.013 [file elife05808s001.zip › spectra/C-NMR_Comp7.pdf]

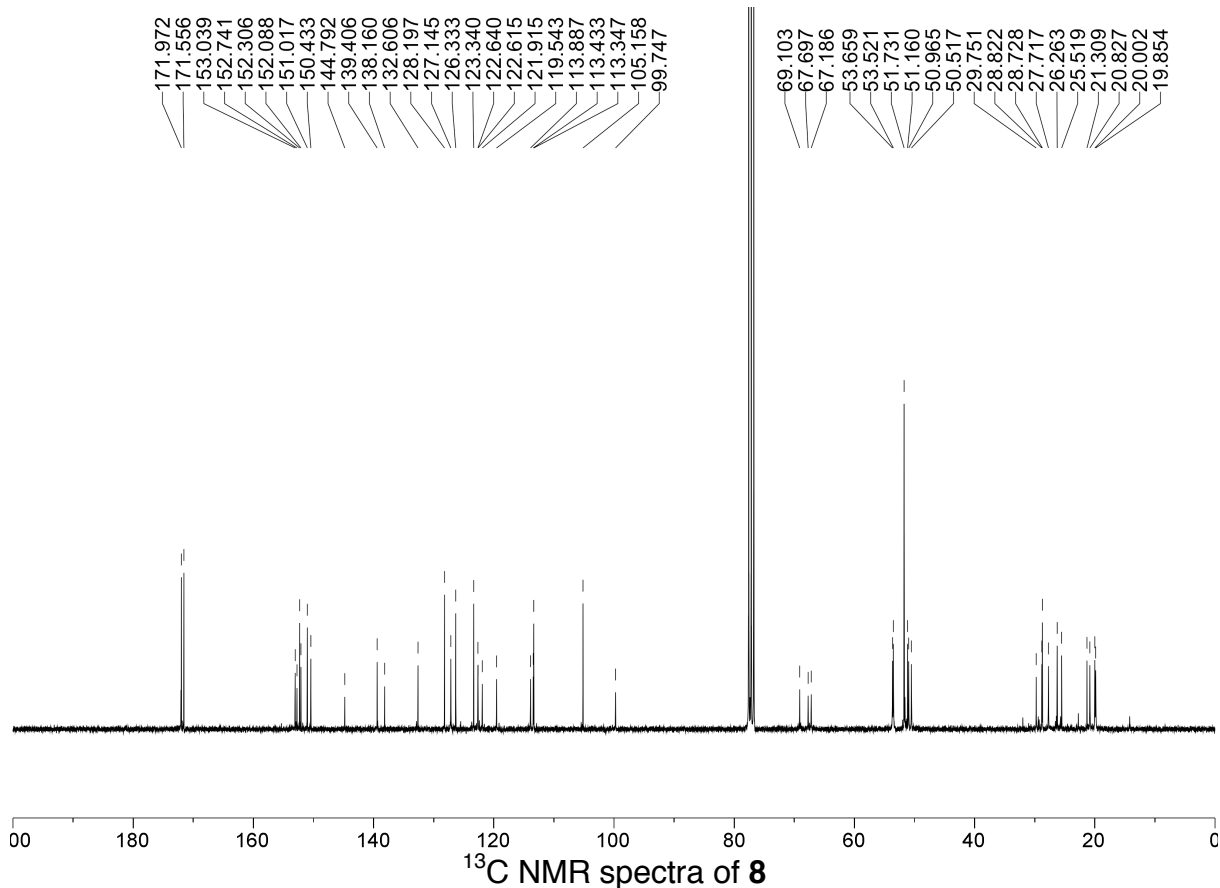

Supplement: Supplementary file 1. — Spectra (NMR and mass). DOI: http://dx.doi.org/10.7554/eLife.05808.013 [file elife05808s001.zip › spectra/C-NMR_Comp8.pdf]

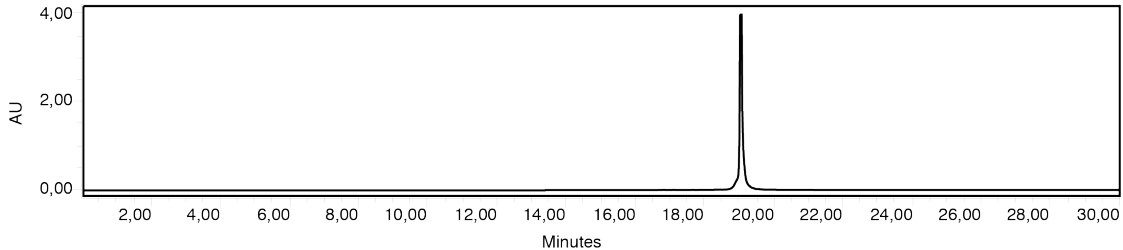

Chromatogram for purification of compound 10

Supplement: Supplementary file 1. — Spectra (NMR and mass). DOI: http://dx.doi.org/10.7554/eLife.05808.013 [file elife05808s001.zip › spectra/CG_Comp10.pdf]

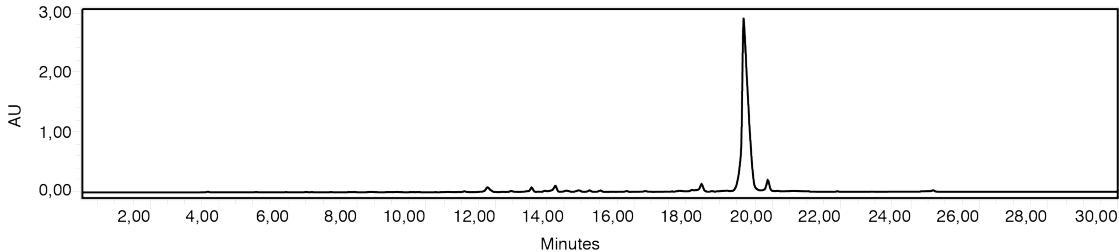

Chromatogram for purification of compound 8

Supplement: Supplementary file 1. — Spectra (NMR and mass). DOI: http://dx.doi.org/10.7554/eLife.05808.013 [file elife05808s001.zip › spectra/CG_Comp8.pdf]

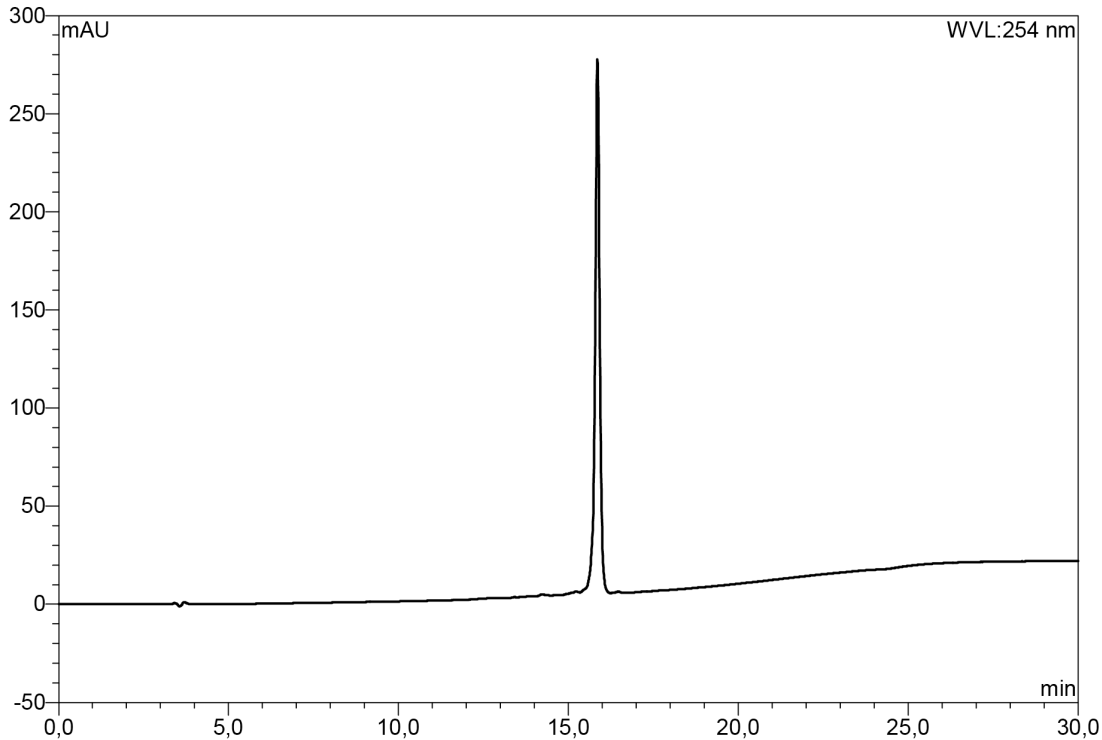

Chromatogram for purification of compound 9

Supplement: Supplementary file 1. — Spectra (NMR and mass). DOI: http://dx.doi.org/10.7554/eLife.05808.013 [file elife05808s001.zip › spectra/CG_Comp9.pdf]

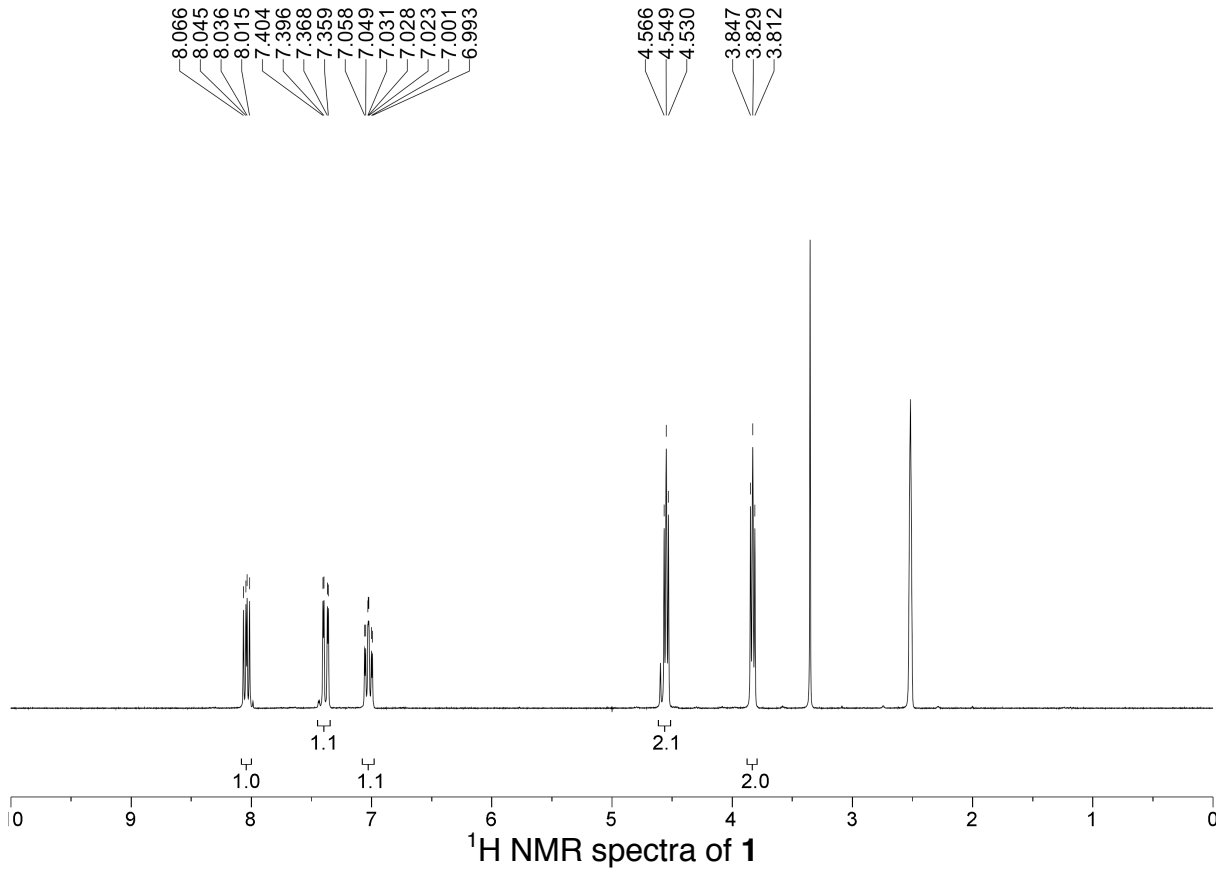

Supplement: Supplementary file 1. — Spectra (NMR and mass). DOI: http://dx.doi.org/10.7554/eLife.05808.013 [file elife05808s001.zip › spectra/H-NMR_Comp1.pdf]

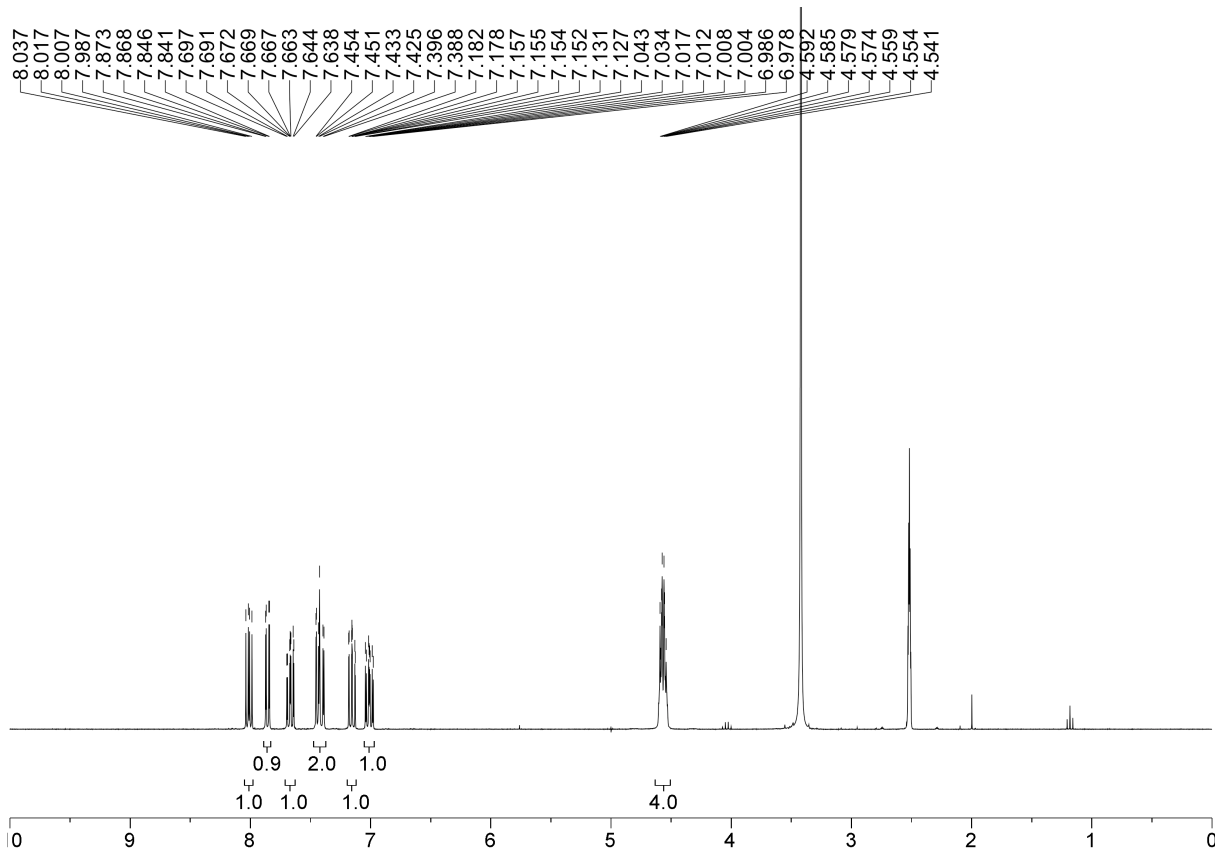

$^1\text{H}$  NMR spectra of **2**

Supplement: Supplementary file 1. — Spectra (NMR and mass). DOI: http://dx.doi.org/10.7554/eLife.05808.013 [file elife05808s001.zip › spectra/H-NMR_Comp2.pdf]

7.900  
7.881  
7.870  
7.854  
7.849  
7.700  
7.695  
7.671  
7.647  
7.642  
7.488  
7.460  
7.183  
7.157  
7.131  
6.664  
6.657  
6.527  
6.519  
6.497  
6.489

4.553  
4.541  
4.537  
4.460  
4.446  
4.435

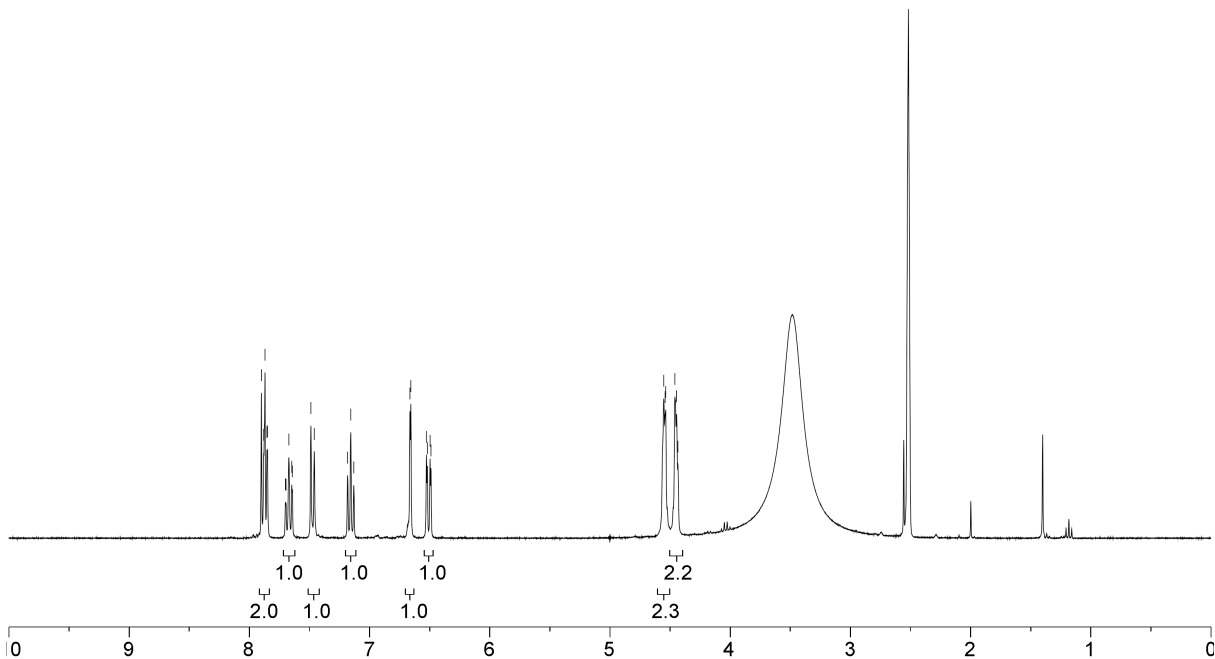

$^1\text{H}$  NMR spectra of **3**

Supplement: Supplementary file 1. — Spectra (NMR and mass). DOI: http://dx.doi.org/10.7554/eLife.05808.013 [file elife05808s001.zip › spectra/H-NMR_Comp3.pdf]

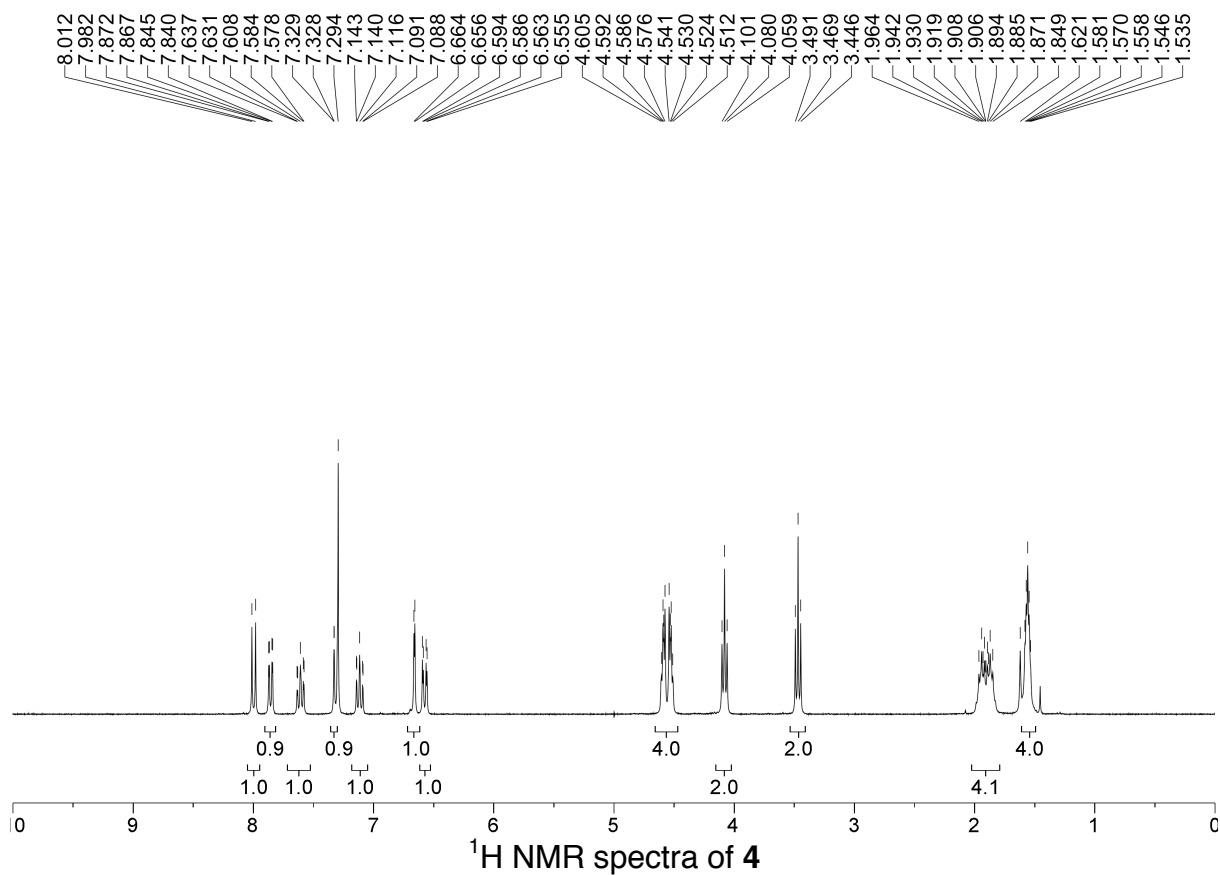

Supplement: Supplementary file 1. — Spectra (NMR and mass). DOI: http://dx.doi.org/10.7554/eLife.05808.013 [file elife05808s001.zip › spectra/H-NMR_Comp4.pdf]

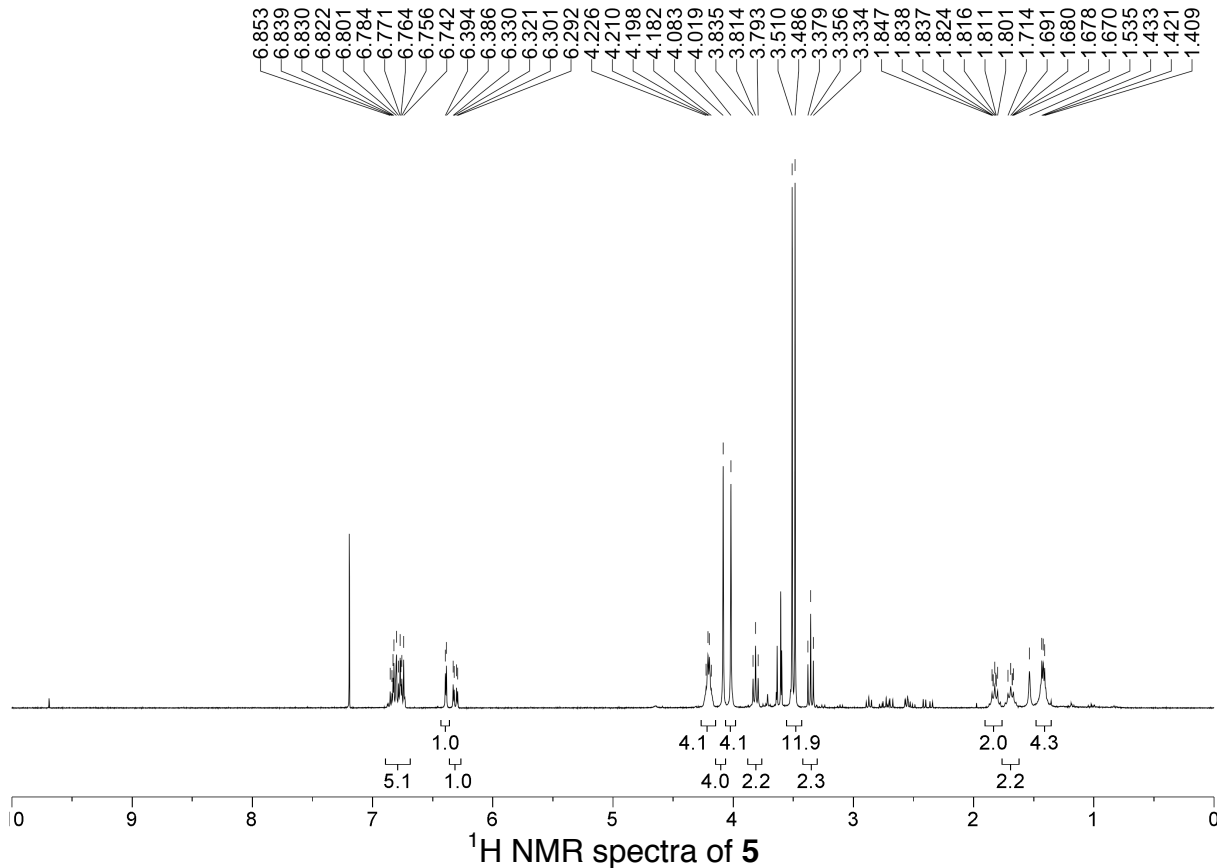

Supplement: Supplementary file 1. — Spectra (NMR and mass). DOI: http://dx.doi.org/10.7554/eLife.05808.013 [file elife05808s001.zip › spectra/H-NMR_Comp5.pdf]

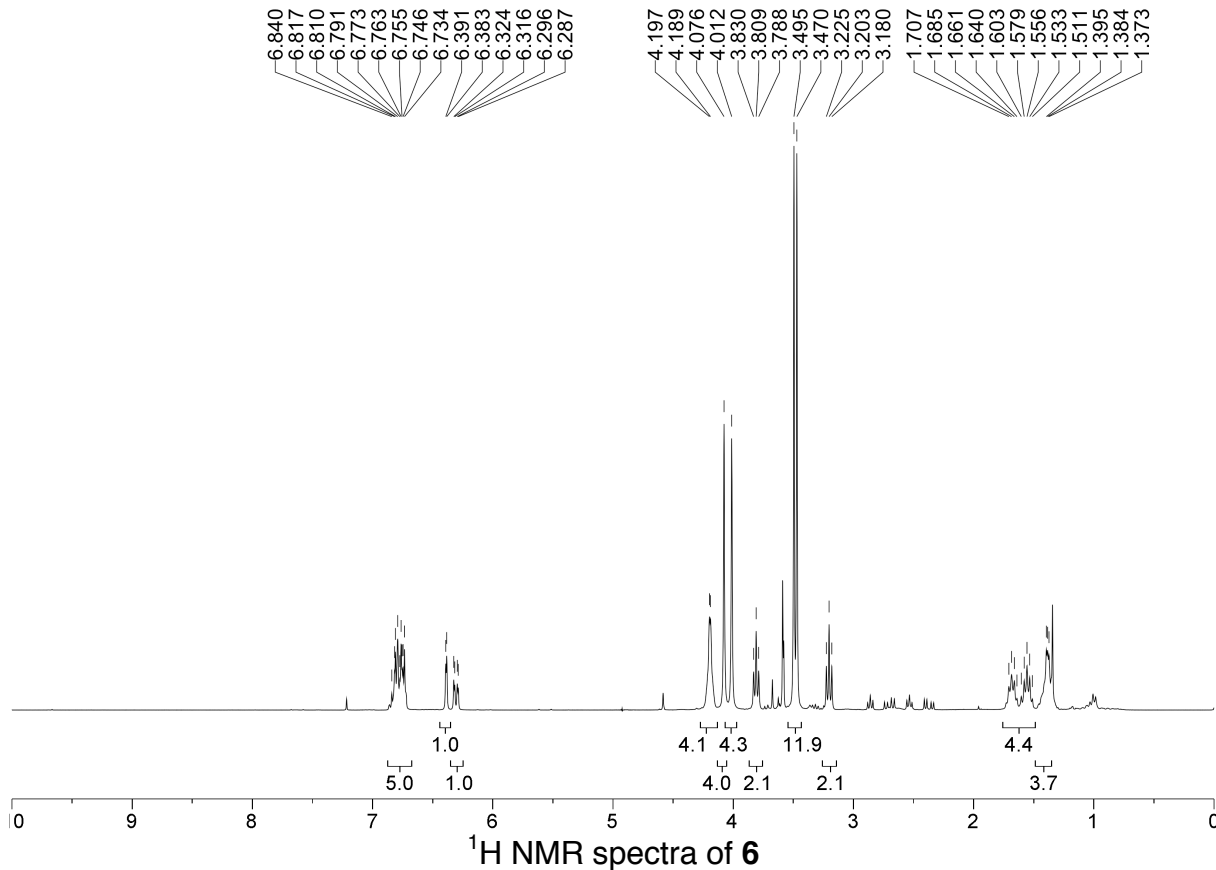

Supplement: Supplementary file 1. — Spectra (NMR and mass). DOI: http://dx.doi.org/10.7554/eLife.05808.013 [file elife05808s001.zip › spectra/H-NMR_Comp6.pdf]

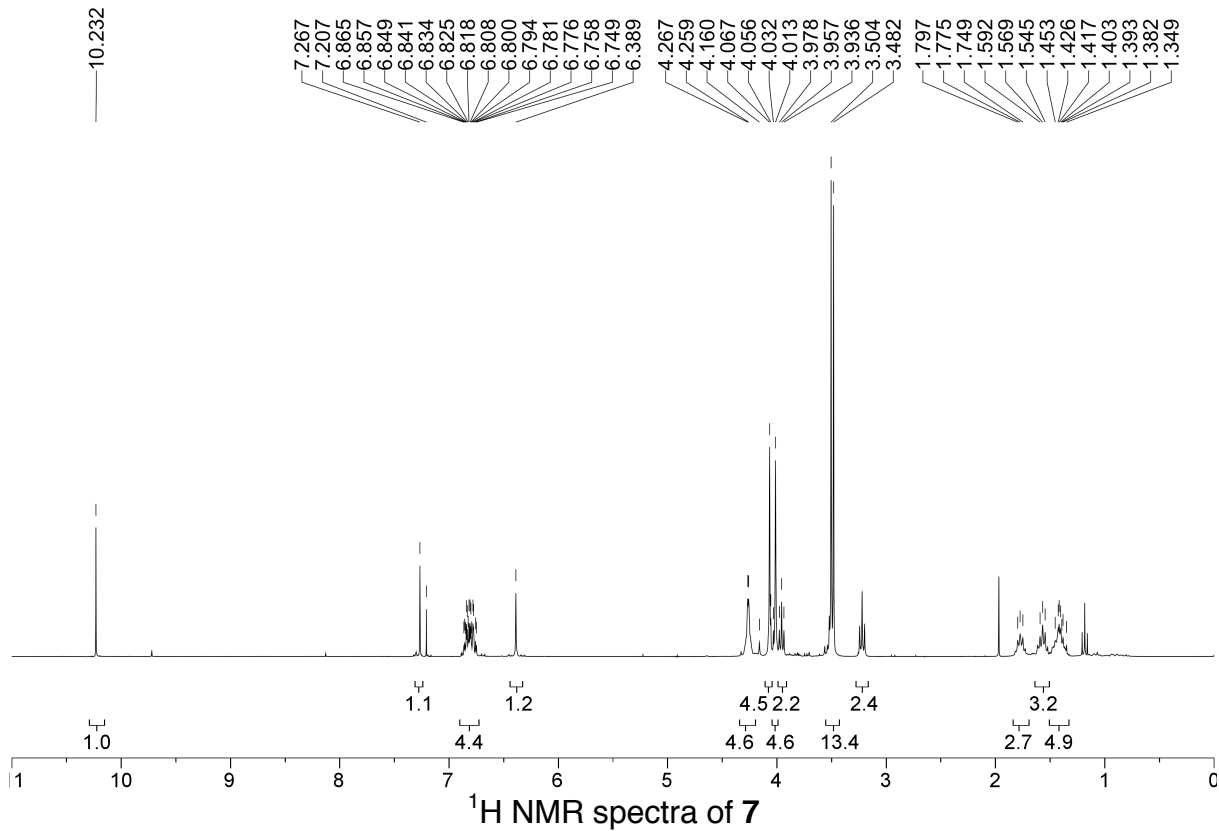

Supplement: Supplementary file 1. — Spectra (NMR and mass). DOI: http://dx.doi.org/10.7554/eLife.05808.013 [file elife05808s001.zip › spectra/H-NMR_Comp7.pdf]

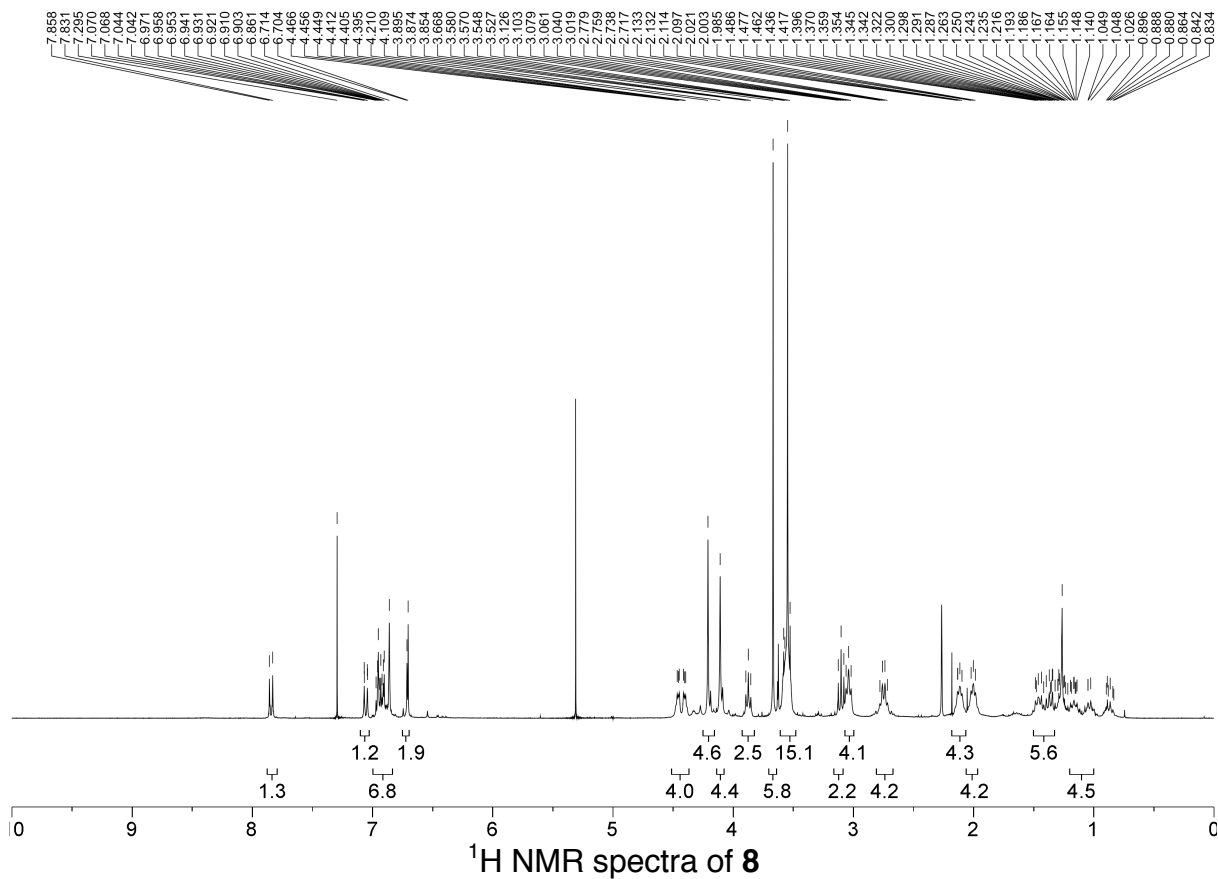

Supplement: Supplementary file 1. — Spectra (NMR and mass). DOI: http://dx.doi.org/10.7554/eLife.05808.013 [file elife05808s001.zip › spectra/H-NMR_Comp8.pdf]

23-Nov-2012 3::9::4

ENS\_AB013 21 (0.572) Cm (17.32)

MeOH+CH<sub>2</sub>Cl<sub>2</sub>

LCT Premier XE KE483

1: TOF MS ES+

7.87e3

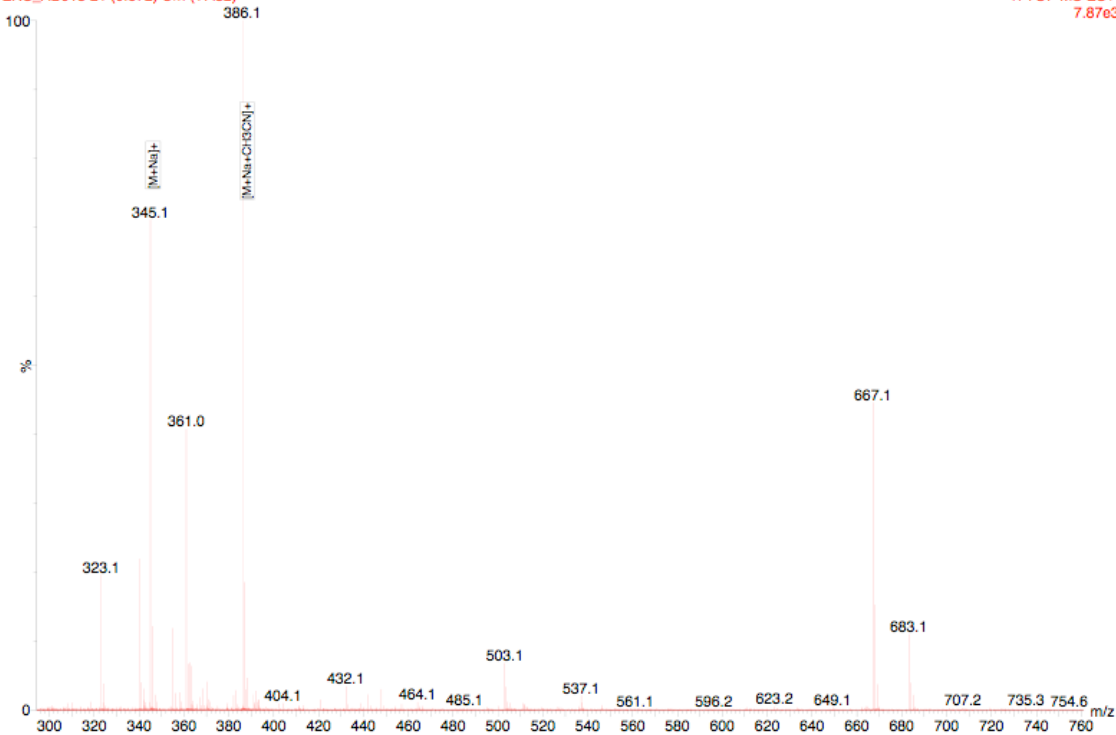

MS Spectra of 2

Supplement: Supplementary file 1. — Spectra (NMR and mass). DOI: http://dx.doi.org/10.7554/eLife.05808.013 [file elife05808s001.zip › spectra/MS_Comp2.pdf]

23-Nov-2012 3:6:6  
ENS\_AB008 23 (0.616) Cm (14:32)

MeOH+CH2Cl2

LCT Premier XE KE483  
2: TOF MS ES-  
7.78e4

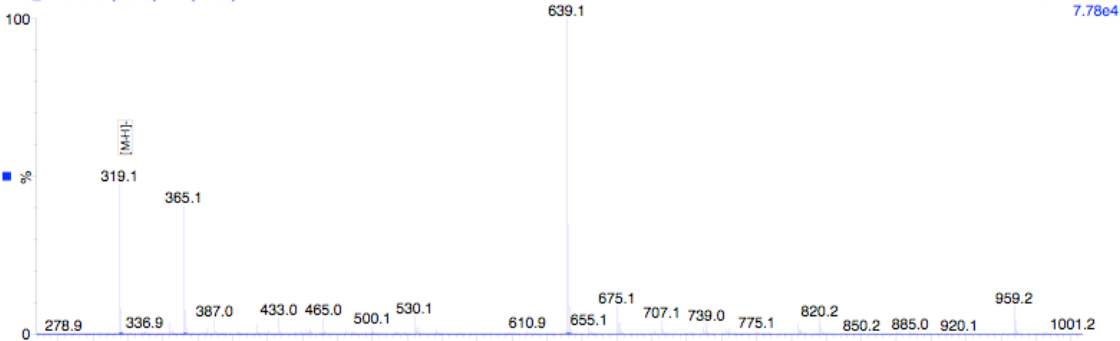

ENS\_AB008 21 (0.573) Cm (17:31)

1: TOF MS ES+  
3.12e4

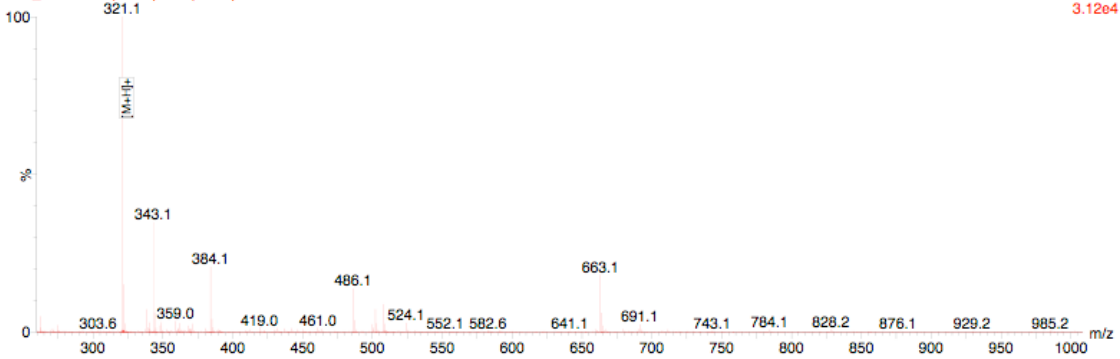

MS Spectra of 3

Supplement: Supplementary file 1. — Spectra (NMR and mass). DOI: http://dx.doi.org/10.7554/eLife.05808.013 [file elife05808s001.zip › spectra/MS_Comp3.pdf]

23-Nov-2012 3::3::7

MeOH+CH<sub>2</sub>Cl<sub>2</sub>

LCT Premier XE KE483

ENS\_AB012 21 (0.572) Cm (17:32)

1: TOF MS ES+

2.52e4

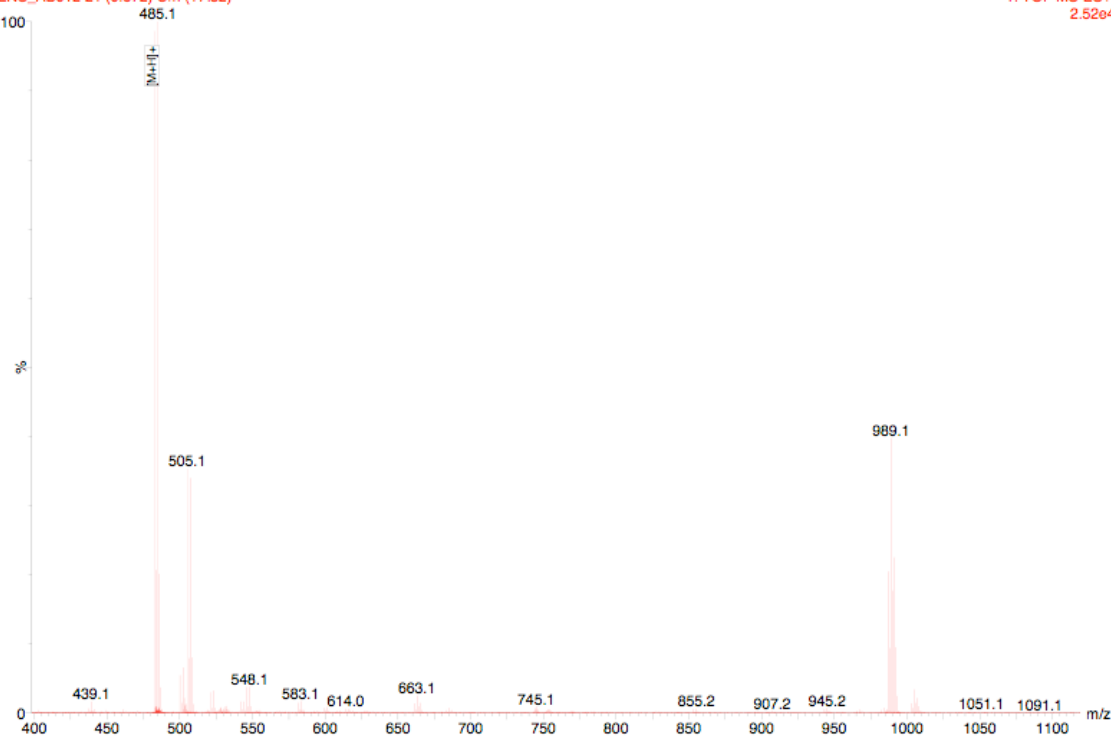

MS Spectra of 4

Supplement: Supplementary file 1. — Spectra (NMR and mass). DOI: http://dx.doi.org/10.7554/eLife.05808.013 [file elife05808s001.zip › spectra/MS_Comp4.pdf]

23-Nov-2012 2::7::6

ENS\_AB028 21 (0.571) Cm (15.39)

MeOH+CH<sub>2</sub>Cl<sub>2</sub>

LCT Premier XE KE483

1: TOF MS ES+

1.61e5

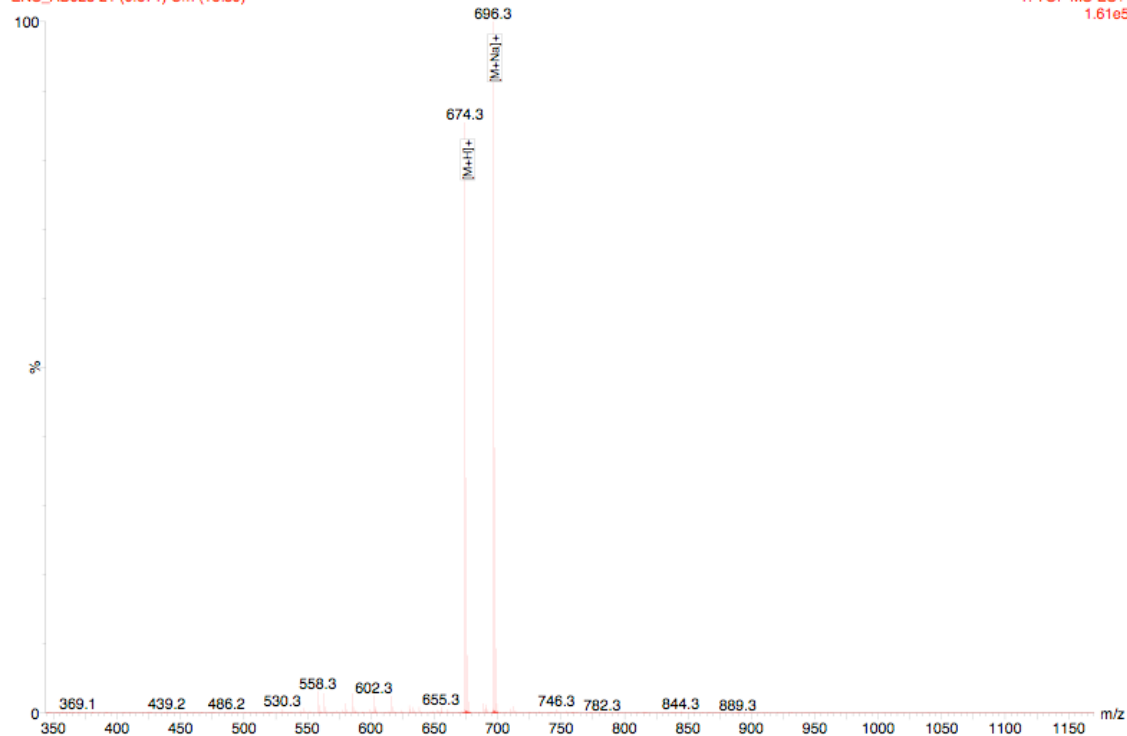

MS Spectra of **6**

Supplement: Supplementary file 1. — Spectra (NMR and mass). DOI: http://dx.doi.org/10.7554/eLife.05808.013 [file elife05808s001.zip › spectra/MS_Comp6.pdf]

23-Nov-2012 2::1::9

MeOH+CH<sub>2</sub>Cl<sub>2</sub>

LCT Premier XE KE483

ENS\_AB031 21 (0.580) Cm (15:33)

2: TOF MS ES-  
2.26e4

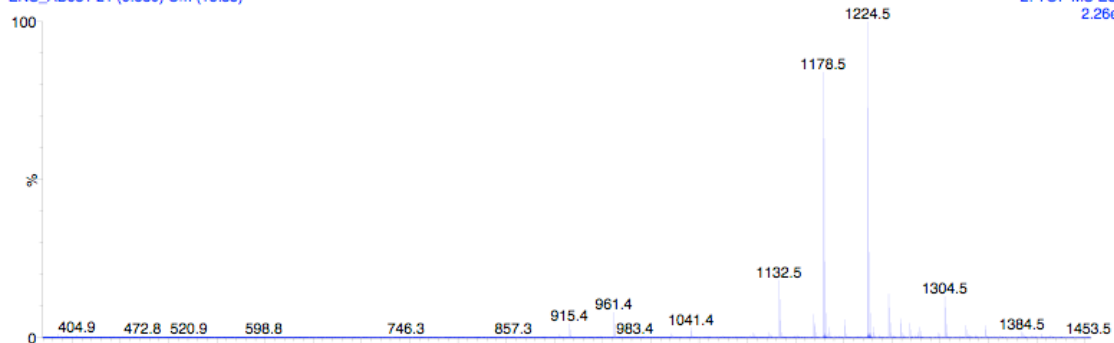

ENS\_AB031 21 (0.572) Cm (17:34)

1: TOF MS ES+  
1.60e5

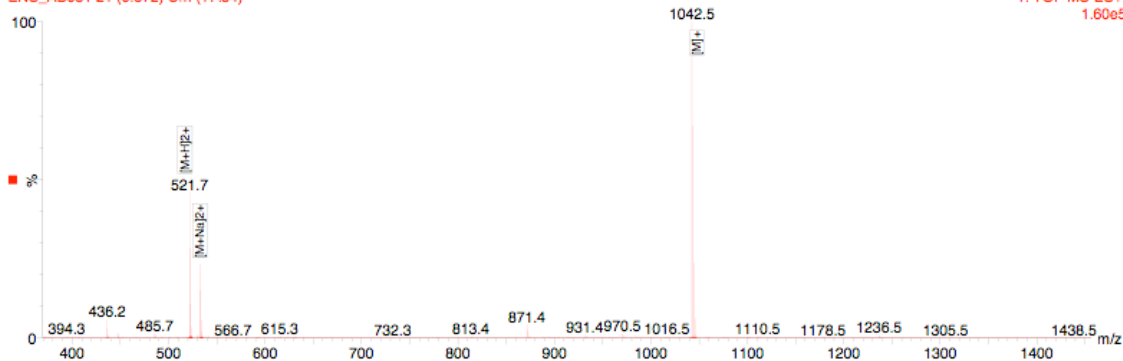

MS Spectra of 8

Supplement: Supplementary file 1. — Spectra (NMR and mass). DOI: http://dx.doi.org/10.7554/eLife.05808.013 [file elife05808s001.zip › spectra/MS_Comp8.pdf]
